# Recovering Marcus Theory Rates and Beyond without the Need for Decoherence Corrections: The Mapping Approach to Surface Hopping

**DOI:** 10.1021/acs.jpclett.3c03197

**Published:** 2024-01-12

**Authors:** Joseph E. Lawrence, Jonathan R. Mannouch, Jeremy O. Richardson

**Affiliations:** † Department of Chemistry and Applied Biosciences, ETH Zurich, 8093 Zurich, Switzerland; ‡ Hamburg Center for Ultrafast Imaging, Universität Hamburg and Max Planck Institute for the Structure and Dynamics of Matter, Luruper Chaussee 149, 22761 Hamburg, Germany

## Abstract

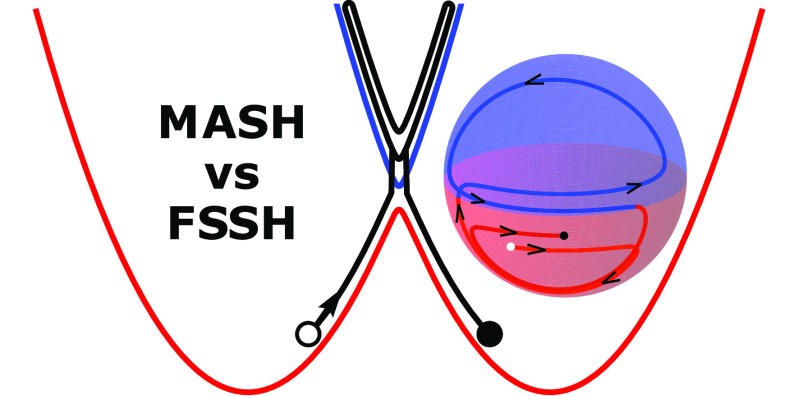

It is well-known that fewest-switches surface hopping (FSSH) fails to correctly capture the quadratic scaling of rate constants with diabatic coupling in the weak-coupling limit, as expected from Fermi’s golden rule and Marcus theory. To address this deficiency, the most widely used approach is to introduce a “decoherence correction”, which removes the inconsistency between the wave function coefficients and the active state. Here we investigate the behavior of a new nonadiabatic trajectory method, called the mapping approach to surface hopping (MASH), on systems that exhibit an incoherent rate behavior. Unlike FSSH, MASH hops between active surfaces deterministically and can never have an inconsistency between the wave function coefficients and the active state. We show that MASH not only can describe rates for intermediate and strong diabatic coupling but also can accurately reproduce the results of Marcus theory in the golden-rule limit, without the need for a decoherence correction. MASH is therefore a significant improvement over FSSH in the simulation of nonadiabatic reactions.

Under the Born–Oppenheimer approximation, one assumes that electronic motion is fast compared to nuclear motion and is therefore adiabatically separated. The resulting picture of nuclei moving on a single adiabatic potential energy surface forms the basis of our modern understanding of molecular structure and dynamics. Despite its great success, there are many important molecular processes for which the Born–Oppenheimer approximation is not valid. Most obviously this can occur in processes, such as photoexcitation, where the electronic degrees of freedom are driven far from equilibrium.^1−6^ However, nonadiabatic dynamics can also occur closer to equilibrium in processes that involve significant redistribution of electron density, such as in electron transfer.^7−11^ The importance of both light–matter interaction and electron-transfer processes to physics, chemistry, and biology as well as modern technology makes the development of practical simulation methods for nonadiabatic dynamics of utmost importance.^12−14^

Unfortunately, finding an exact solution of the full coupled electron–nuclear Schrödinger equation is impractical for most systems of interest, and hence, approximations need to be made.^15−19^ Fortunately, however, the relatively high mass of atomic nuclei means that it is often a reasonable approximation to treat them as classical particles with well-defined positions and momenta. In 1990, Tully proposed what has become the most widely used of such “mixed quantum–classical” methods for simulating nonadiabatic processes, known as fewest-switches surface hopping (FSSH).^20^ Within FSSH, the nuclei predominantly move under the force of a single adiabatic potential energy surface with occasional stochastic hops between the surfaces. The probabilities for these hopping events are determined on the basis of the evolution of the electronic wave function under the time-dependent Hamiltonian generated by the nuclear trajectory.

Fewest-switches surface hopping has been successfully applied to study a wide range of nonadiabatic processes.^3−6^ However, it has long been appreciated that there are problems that lead to a breakdown in the assumptions behind the FSSH algorithm.^21−24^ The result is a deviation between the number of trajectories on each surface and the wave function coefficients, which can therefore be termed an inconsistency error. At a more fundamental level, the error can be attributed to a failure to describe the decoherence of the electronic wave function that results from the splitting of a wavepacket after passing through a coupling region.^21−23^ This observation has led to the introduction of many different ad hoc decoherence corrections, aimed at fixing the inconsistency (overcoherence) error of FSSH.^23−29^

Due to their ad hoc nature, decoherence corrections are not guaranteed to consistently improve the results of a calculation.^30^ However, one area in which they have been shown to be essential is processes, such as electron transfer, which involve slow population transfer in strongly nonadiabatic systems (weak diabatic coupling, Δ). A series of papers from Subotnik and co-workers has demonstrated that the standard FSSH algorithm fails to properly describe the Δ^2^ scaling of the rate predicted by Fermi’s golden rule and the famous Marcus theory of electron transfer.^31−35^ This was explained in terms of repeated crossings of the nonadiabatic coupling region, leading to a buildup of the inconsistency error.^31^

Recently, an alternative to FSSH has been derived known as the mapping approach to surface hopping (MASH).^36^ MASH was designed to offer the best of both worlds between surface hopping and mapping approaches, such as the Meyer–Miller–Stock–Thoss mapping^37,38^ and spin mapping.^39,40^ Unlike FSSH, which was proposed heuristically, MASH can be rigorously derived from the quantum–classical Liouville equation (QCLE).^41−46^ Tests against exact results for the Tully models, a series of spin-boson models, as well as 3-mode and 24-mode vibronic models of pyrazine have shown that the results of MASH are generally as good as or better than those of FSSH for an equivalent computational cost.^36^ Perhaps most interesting are the results for the spin-boson model, where the system crosses the coupling region many times during the dynamics. One might have expected that decoherence corrections were necessary to improve upon the FSSH results. However, MASH shows a significant improvement even without the addition of decoherence corrections. This raises the question: how well will MASH perform in systems exhibiting slow population transfer with weak diabatic coupling where the errors of FSSH are known to be particularly pronounced?^31^

In the following, we will attempt to answer this question. In doing so, we will explore the difference between MASH and FSSH in terms of the language of decoherence, revisiting the reasons for the breakdown of FSSH in systems with weak diabatic couplings and showing how MASH improves upon these issues. We will begin by giving an overview of the two methods, highlighting the key similarities and differences between the FSSH and MASH algorithms. We will then describe how to simulate nonadiabatic rates using these approaches before a detailed discussion of how each of the methods performs for a range of different physically relevant parameter regimes.

## Methods.

 Here we give a brief description of the two methods in the case of a two-level system. Both FSSH and MASH treat the nuclear motion classically, with the nuclear positions and momenta represented by the classical variables **q**(*t*) and **p**(*t*), respectively. Between hopping events, the nuclei evolve under a force that is given by the derivative of the adiabatic potential corresponding to the “active surface”
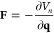
1where *n* is the active-state variable, and we label the upper adiabat *+* and the lower adiabat −. Electronic wave function coefficients, *c*_±_(*t*), are then propagated according to the time-dependent Schrödinger equation under the Hamiltonian generated by the nuclear trajectory. In both theories, these coefficients are used to determine when to hop but are not used to calculate adiabatic population observables, which are instead obtained directly from the fraction of trajectories on a given active surface.^4^ An intuitive picture of the electronic dynamics can be obtained using the coordinates of the Bloch sphere
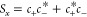
2a
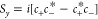
2b
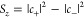
2cThis highlights the equivalence of the electronic dynamics to the rotation of a classical spin around a magnetic field
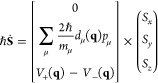
3where *V*_±_ are the potentials corresponding to adiabatic states ϕ_±_ and  is the nonadiabatic coupling vector.

What differs between FSSH and MASH is how the hops between the surfaces are determined. Within FSSH, the probability of hopping from one surface to the other in a time step *δt* is given by
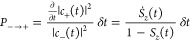
4a
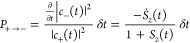
4bwhere negative probabilities indicate no hop. In contrast to this, the active surface in MASH is obtained deterministically by the simple condition
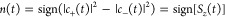
5i.e., the active state is the one with the larger probability, |*c*_±_(*t*)|^2^. The fact that MASH is deterministic might seem surprising, particularly given that it is the stochastic nature of FSSH that allows it to describe wavepacket splitting. However, as in other mapping-based methods,^39^ the stochastic nature of surface hopping is replaced in MASH by sampling over initial wave function coefficients, as we shall explain below. To complete the specification of the dynamics, we need to define what happens to the momentum at a hopping (or attempted hopping) event. While there has been some debate in the literature as to how this should be done in FSSH,^47,48^ the derivation of MASH from the QCLE leads to a unique prescription for how to deal with momentum rescaling and so-called frustrated hops (where the trajectory has insufficient energy to hop). The result is equivalent to what was originally advocated by Tully^49^ (along with many others^50,51^). The momenta are rescaled along the direction of the nonadiabatic coupling and are reflected in all cases in which they do not have sufficient energy to hop.

This suffices to describe the dynamical evolution of MASH and FSSH; however, there is one additional important difference, how the simulation is initialized. For ease of comparison between FSSH and MASH, we will focus here on the calculation of correlation functions that involve only adiabatic populations and nuclear configurations (although we note that the MASH derivation leads to a rigorous prescription for the calculation of correlation functions involving electronic coherences). For a system starting in a specific adiabatic state, both FSSH and MASH are initialized with the corresponding active state, *n*(0). In FSSH, the wave function coefficients are initialized as the corresponding pure state; e.g., if the initial state is *n* = + then *c*_+_(0) = 1 and *c*_–_(0) = 0 and the initial **S** vector points to the north pole of the Bloch sphere. In contrast, the wave function coefficients in MASH are sampled such that the initial **S** is distributed over the entire hemispherical surface of the Bloch sphere corresponding to the initial state, with a probability density proportional to |*S*_*z*_|.^a^ It is this sampling that effectively replaces the stochastic nature of the hops in FSSH.

A discussion of surface hopping would not be complete without covering decoherence corrections. Importantly, MASH has the additional property that its decoherence corrections can be rigorously derived. Because MASH is an exact short-time approximation of the QCLE, it can be systematically improved toward the full QCLE result by application of so-called “quantum jumps”.^36^ These jumps differ from decoherence corrections in that they cannot be applied too often (i.e., no quantum Zeno effect). In general, however, quantum jumps increase the cost of the simulation. The exception to this is if the quantum jump is applied at a point where there is negligible coherence between the two adiabatic surfaces, i.e., ⟨*S*_*x*_⟩ = ⟨*S*_*y*_⟩ = 0. At such points, the quantum jump is equivalent to resampling the **S** vector from the hemisphere corresponding to the current active state with the |*S*_*z*_| probability density (equivalent to the initial sampling). This is the MASH decoherence correction.^36^ It is analogous to the FSSH decoherence correction, where one resets the wave function coefficients as the pure state corresponding to the current active state. As it can be understood as a special case of a quantum jump, it is safe to use and rigorously justified when applied in regions where ⟨*S*_*x*_⟩ = ⟨*S*_*y*_⟩ = 0, i.e., far from the regions of nonadiabatic coupling. Crucially, as we shall see, MASH is more accurate than FSSH at short to intermediate times; hence, one can often afford to wait until this condition is satisfied before applying a correction to the dynamics (and in many cases one may not even need to correct the dynamics at all).

*Rate Calculations.* Full details of the calculation of rate constants with MASH and FSSH are discussed in the Supporting Information. Here we give an overview of the most important aspects of reaction rate theory, focusing on the advantages of MASH over FSSH in two key areas: efficiency and accuracy.

Typically, the accurate determination of rate constants from a direct simulation of the population dynamics is not possible, as the barrier crossing is a rare event, and prohibitively long trajectories would be required to observe a statistically significant number of reactions. The standard approach used to overcome this problem is the flux-correlation formalism.^52^ This avoids the rare-event problem by reformulating the rate in terms of a correction to transition-state theory, the transmission coefficient. Importantly, the calculation of the transmission coefficient involves running only a short simulation up to the “plateau” time, *t*_pl_, which is much shorter than the time scale of the reaction (*t*_pl_ ≪ τ_rxn_) but long enough that the initial transient behavior has subsided and the population decay is exponential.^52^

Unfortunately, the FSSH dynamics do not obey time-translation symmetry, and hence, the flux-correlation formalism does not rigorously give the same result as calculating the rate from direct population dynamics. A number of approaches for overcoming this issue have been suggested, such as using initial wave function amplitudes in the flux-correlation function generated from approximate backward-propagation schemes,^34,53^ as well as the use of dynamically enhanced sampling in the form of forward-flux sampling.^54^ Here, to avoid making further approximations, we simply calculated the FSSH rate from direct population dynamics, which can be achieved due to the low computational cost of the model employed. The calculation of reaction rates with MASH presents a significant advantage in this regard. The dynamics of MASH do rigorously obey time-translation symmetry. This means that all of the usual machinery of the flux-correlation formalism (such as the Bennett–Chandler method^55−57^) can be used to improve the efficiency of rate calculations in a way that is rigorously equivalent to the rate that would be obtained (less efficiently) with a direct simulation of the population dynamics.

The second difficulty associated with the calculation of reaction rates with FSSH is the overcoherence error.^31−35^ This error is known to occur in problems where the system passes through regions of strong nonadiabatic coupling (equivalent to weak diabatic coupling) multiple times, resulting in an active state that is inconsistent with the wave function coefficients. Importantly, the dynamics of MASH can never become inconsistent in the way they do in FSSH, as the active state is determined explicitly from the wave function coefficients. This means that one may expect the overcoherence error to be less significant in MASH than in FSSH. To assess this, we consider the MASH and FSSH dynamics in two different regimes. First, we focus on how the error affects dynamics near the plateau time (*t* ∼ *t*_pl_). This is done by calculating rates from the slope of the product population ⟨*P*_p_(*t*)⟩ after the initial transient behavior has subsided for a system initialized in the reactant well in a classical thermal distribution. Second, we consider the dynamics over the time scale of the reaction (*t* ∼ τ_rxn_) by simulating the full population decay.

*Model.* To compare numerically the accuracy of MASH and FSSH for the calculation of nonadiabatic rates, we consider the prototypical model for electron transfer, the spin-boson model.^58^ For ease of interpretation, we will consider the Brownian-oscillator form of the spin-boson model,^59−62^ which consists of a harmonic (mass-weighted) solvent polarization coordinate, *Q*, and an Ohmic bath describing the effect of friction along *Q*, with spectral density *J*(ω) = *γω*. The diabatic potentials along the solvent polarization coordinate are then the famous Marcus parabolas^8^
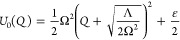
6a
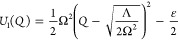
6bwhere Λ is the Marcus reorganization energy, ε is the reaction driving force, and Ω is the characteristic frequency of the parabola. The two diabatic states are coupled by a constant diabatic coupling Δ, and the resulting adiabatic potentials along the solvent polarization coordinate are given by
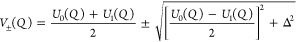
7In both FSSH and MASH simulations, the initial positions and momenta are sampled from the classical Boltzmann distributions (not Wigner functions), and the initial active state is chosen with the associated Boltzmann weighting. As the nuclei are classical, the coupling of the solvent polarization coordinate, *Q*, to its environment can be implemented efficiently using a Langevin equation with friction coefficient γ. Note this is formally equivalent to explicitly simulating the full multidimensional bath.^59−62^

In the limit of weak diabatic coupling (Δ → 0), Marcus theory predicts that the rate for going from one well to the other is given by^8^
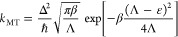
8where β = 1/*k*_B_*T* is the inverse temperature. Importantly, Marcus theory is exact for this model in the weak-coupling limit under the assumption that the nuclear motion can be treated classically, i.e., in the absence of nuclear quantum effects such as zero-point energy and tunneling. This makes Marcus theory a very useful benchmark for assessing the accuracy of FSSH and MASH, which also assume that the nuclear motion can be treated classically. To assess their behavior for intermediate values of Δ, where Marcus theory is not applicable, numerically exact quantum-mechanical rates were calculated using the hierarchical equations of motion (HEOM).^63,64^ All HEOM calculations were performed using the HEOM-Lab code^65,66^ following the method described in refs (62) and (67).

For both MASH and FSSH, the long-time behavior of ⟨*P*_p_(*t*)⟩ is independent of the precise definition of reactants and products.^b^ However, the definition of reactants and products will affect its short-time behavior. The optimum choice for the calculation of rates is the one for which the dynamics of a system initialized in the reactants most quickly settles into an exponential decay. Normally, this is a purely practical matter; however, choosing a (nearly) optimal definition has additional importance in the present study: it allows us to separate the short- and long-time errors. The definition we use is that everything on the lower adiabatic surface to the right of the diabatic crossing or on the upper adiabatic surface on the left of the diabatic crossing is the product and vice versa for the reactant. Mathematically, this corresponds to
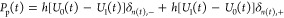
9where *h*(*x*) is the Heaviside step function and *P*_r_ = 1 – *P*_p_. This definition works well for all of the cases considered in this work. We demonstrate numerically in the Supporting Information that this gives the same rate constants as a purely position-space definition in the normal regime, or a purely adiabatic definition in the inverted regime, while having a shorter transient.

The parameters for the model are taken to be *βΛ* = 12, βℏΩ = ^1^/_4_, and γ = Ω, for a range of values of ε and Δ. These parameters were chosen to allow a clear comparison of the accuracy of MASH and FSSH, at a reasonable computational cost. In particular, the reorganization energy was chosen to be sufficiently high that the population transfer is in the slow incoherent limit but sufficiently low that it is possible to run direct population dynamics. This allows us to directly calculate FSSH rates, without needing to employ backward propagation or forward-flux sampling. Additionally, it allows us to demonstrate numerically that in MASH direct population dynamics are equivalent to the results obtained using the flux-correlation formulation, which we show in the Supporting Information. The characteristic frequency was chosen to make the system as classical as possible without the HEOM calculations becoming too expensive. This was done as our focus here is on assessing the relative accuracy of the dynamics of MASH and FSSH, rather than the importance of the nuclear quantum effects. Finally, it is known that the effect of overcoherence error becomes less pronounced at high friction,^35^ and hence, to make the test of MASH as stringent as possible, we consider a system in the underdamped γ < 2Ω regime. Systems with a larger reorganization energy and a higher friction are considered in the Supporting Information.

**Figure 1:**
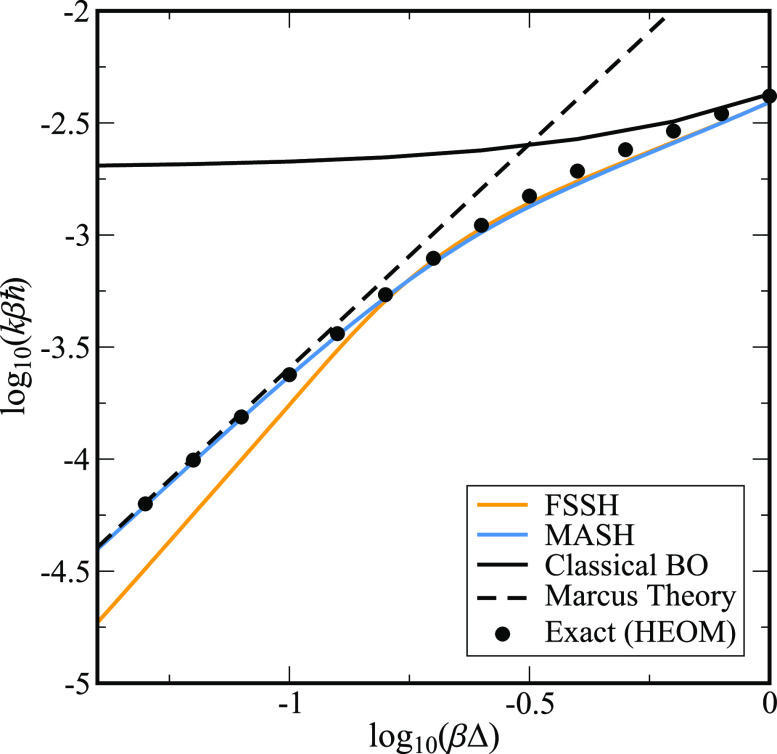
Log–log plot of the rate vs the diabatic coupling for a symmetric, *βε* = 0, spin-boson model, with βℏΩ = ^1^/_4_, γ = Ω, and *βΛ* = 12. FSSH and MASH rates were calculated from the slope of ⟨*P*_p_(*t*)⟩ at the plateau time, between *t* = 10βℏ and *t* = 20βℏ. Note that Figure S4 shows similar results for an asymmetric model.

*Results and Discussion.*Figure 1 compares the rates calculated at the plateau time for a symmetric reaction, ε = 0, as a function of the diabatic coupling, Δ. We see that, for intermediate to large values of diabatic coupling, log_10_(*βΔ*) ≳ −0.75, MASH, FSSH, and HEOM all closely agree, with the HEOM rate showing only a slight ∼10% enhancement due to shallow tunneling. For smaller values of Δ, the reaction approaches the golden-rule regime, where Marcus theory is valid. Here we see that MASH continues to closely match the exact results predicted by HEOM, while FSSH begins to deviate significantly with an unphysical slope. This deviation is consistent with previous observations that FSSH struggles in this limit due to its overcoherence error.^31−34^ However, it raises the question of why MASH does not show a similar error.

**Figure 2:**
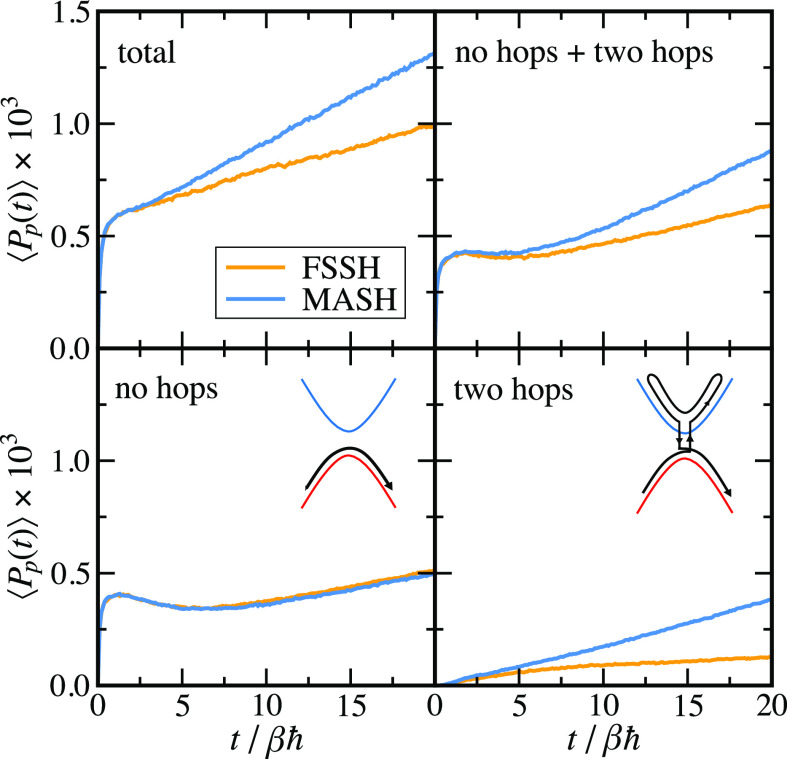
Decomposition of the population of products according to the number of hops for a symmetric, *βε* = 0, spin-boson model, with βℏΩ = ^1^/_4_, γ = Ω, and *βΛ* = 12 in the limit of weak diabatic coupling, log_10_(*βΔ*) = −^7^/_5_.

To understand this, in Figure 2 we analyze ⟨*P*_p_(*t*)⟩ for the smallest value of Δ considered in Figure 1. The top left panel of Figure 2 shows the full ⟨*P*_p_(*t*)⟩. Although MASH and FSSH agree during the initial transient, the slope after this time differs significantly, with FSSH predicting a much slower population transfer. The remaining panels decompose ⟨*P*_p_(*t*)⟩ into contributions from trajectories that have hopped zero or two times between time zero and the current time, *t*. The top right panel shows the sum of the zero- and two-hop trajectories. We see that the difference in the slopes of the MASH and FSSH curves closely resembles those in the full ⟨*P*_p_(*t*)⟩, implying that other terms are contributing to only the transient and not the rate. Hence, to understand the difference between the MASH and FSSH rates, one can focus on just these trajectories. Unsurprisingly, the no-hop contribution to ⟨*P*_p_(*t*)⟩ (which involves just a single passage through the crossing region) agrees very closely between MASH and FSSH. The key difference occurs in the trajectories that hop twice. The contribution of these trajectories, along with a depiction of a corresponding typical reactive path, is shown in the bottom right panel. From this, we see that trajectories that hop twice contribute significantly (and correctly) to the rate in MASH but contribute only a very small amount in FSSH. Hence, the rate predicted by FSSH can be expected to be up to a factor of 2 too small, as previously pointed out by Jain and Subotnik in ref (34).

**Figure 3:**
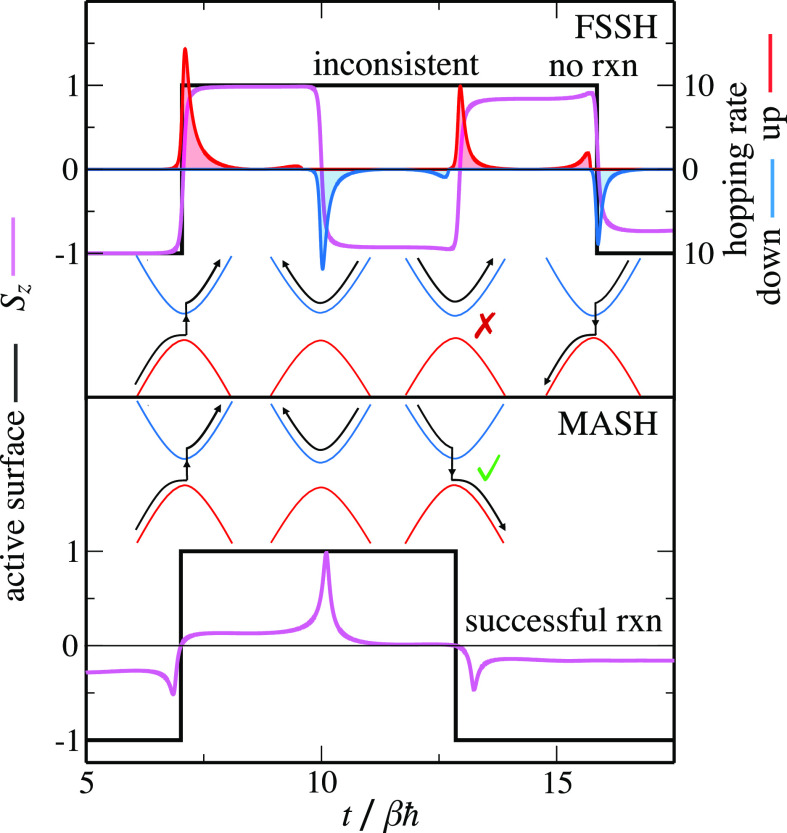
Example of a typical incorrect “two-hop” FSSH trajectory that fails to react, along with a comparable but correct MASH trajectory. The problem for FSSH occurs on the third crossing, where the wave function is inconsistent with the active state. *S*_*z*_ is then predominantly moving up, meaning that the probability of hopping down is almost zero. This example is taken from a calculation with βℏΩ = ^1^/_4_, γ = 0, *βε* = 0, and *βΛ* = 12.

Having established that it is the two-hop trajectories that differ between MASH and FSSH, we still need to explain why these trajectories go wrong in FSSH but not in MASH. Figure 3 illustrates the behavior of a typical two-hop trajectory in FSSH that “should” react but does not. The trajectory starts in the reactant well at time zero. When *t* ≈ 7βℏ, the trajectory reaches the crossing and hops up due to the strong nonadiabatic coupling and correspondingly large hopping probability. Having hopped up, the trajectory then continues on the upper state before turning around and coming back toward the avoided crossing. Note that at this point the trajectory is not significantly affected by inconsistency or overcoherence error, as the wave function coefficients are essentially still in a pure state corresponding to the active surface (i.e., *S*_*z*_ ≈ 1). When *t* ≈ 10βℏ, the trajectory passes through the avoided crossing for a second time, and most trajectories hop down (returning to the reactants). However, we follow one of the few that remain on the upper surface (probability ∝Δ^2^). Now the wave function (which is predominantly in the lower state, *S*_*z*_ ≈ −1) is inconsistent with the active surface. When the trajectory returns to the avoided crossing for a third time, we expect it to hop down to the product well. However, the wave function is evolving in the opposite direction to the expected hop (from down to up instead of up to down). Hence, the probability of jumping down is almost zero, and the trajectory incorrectly stays on the upper surface, leading to no reaction. In contrast, MASH trajectories cannot have this problem. When an equivalent MASH trajectory approaches the avoided crossing for the third time, its spin vector is guaranteed to correctly point up (because of the consistency between its spin vector and the active surface). On passing through the crossing region, its spin vector will then flip down to the lower hemisphere, resulting in a downward hop and a successful reaction.

**Figure 4:**
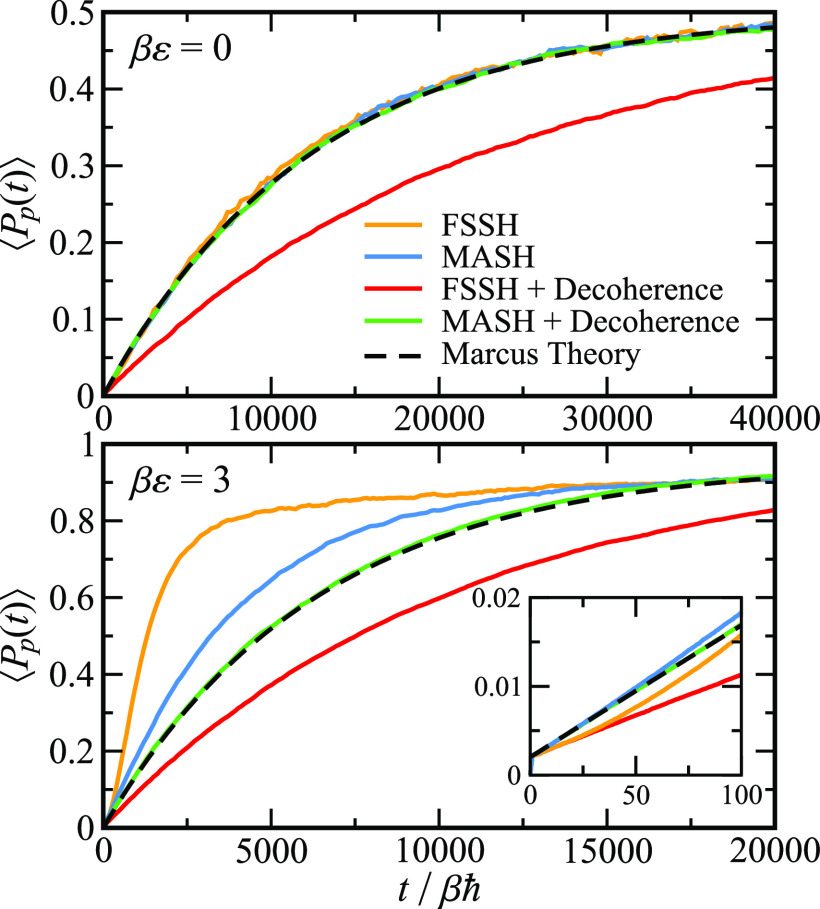
Full population decay for symmetric, *βε* = 0, and asymmetric, *βε* = 3, spin-boson models, with βℏΩ = ^1^/_4_, γ = Ω, and *βΛ* = 12 in the limit of weak diabatic coupling, log_10_(*βΔ*) = −^7^/_5_. The inset shows ⟨*P*_p_(*t*)⟩ at short to intermediate times. Decoherence corrections are applied only when the energy gap is large (*V*_+_ – *V*_–_ > 4*k*_B_*T*), making long-time behavior consistent with short to intermediate time. This illustrates not only that MASH is more accurate than FSSH without the application of decoherence corrections but also that, unlike in FSSH, simple decoherence corrections are sufficient to bring MASH into line with the correct result. Note that the addition of the decoherence correction does not affect the results depicted in Figure 1, as demonstrated in Figure S8.

So far, we have considered only the dynamics on the time scale of a single barrier crossing. However, in the limit of weak diabatic coupling, the system may come back to the diabatic crossing (the region of large nonadiabatic coupling) many times before the reaction takes place. This can lead to a buildup of overcoherence error, causing the long-time rate behavior to deviate significantly from the short-time behavior. To investigate this effect, Figure 4 shows the population of products, ⟨*P*_p_(*t*)⟩, for the full population decay, for two different driving forces, *βε* = 0 and *βε* = 3 = *βΛ*/4, with all other parameters kept the same as in Figure 2.

Considering first the top panel of Figure 4, where *βε* = 0, we see immediately that the long-time behavior of both MASH and FSSH agrees perfectly with the Marcus theory. This is a surprising result, as on the basis of the short-time behavior we would expect FSSH to be too slow. However, it can be explained away as a fortuitous cancellation of errors due to the symmetry of the model when ε = 0. This assumption is confirmed by considering the behavior of an asymmetric reaction, *βε* = 3, as shown in the bottom panel. The short-time behaviors of the symmetric and asymmetric systems are similar as one can see from the inset.^c^ At long times, however, we see that for the asymmetric system there is no fortuitous cancellation of errors. Instead, the buildup of overcoherence error in FSSH leads to a population decay that is noticeably too fast, with a half-life approximately 3.5 times shorter than that of Marcus theory. In contrast, MASH goes from being almost exact at short times to being ∼1.4 times too fast at long times.^d^ We see, therefore, that while both MASH and FSSH suffer from a buildup of overcoherence error at long times, this error is significantly more pronounced in FSSH.

The buildup of overcoherence error at long times is of course well established. While it is nice that this error is much smaller in MASH than FSSH, in real simulations on such incredibly long time scales one should apply decoherence corrections in both theories. In this regard, the short-time accuracy of MASH also presents a significant advantage. To see why, we note that the application of decoherence corrections is always a balancing act: you need to apply them often enough to fix the overcoherence error, but apply them too often, and you will force the system to remain forever on the same adiabat (the quantum Zeno effect). The advantage of MASH is that it requires decoherence corrections less often to obtain accurate results. This means that they can be applied only in regions where it is safe to do so, such as the reactant wells, and not in the vicinity of the coupling region. This makes it more robust and means that simpler decoherence schemes can be successfully used.

In Figure 4, we demonstrate this by considering the behavior of MASH and FSSH when one applies a simple decoherence correction.^36^ For the sake of simplicity, we use an energy cutoff such that decoherence corrections are applied only in the reactant and product wells, where it is safe to do so. The actual cutoff used was *V*_+_ – *V*_–_ > 4*k*_B_*T*; however, the results are insensitive to the precise value, provided it is small enough that a decoherence correction is applied when the trajectory is in the well and large enough that it is not applied in the crossing region.^e^ In the top panel, we see that, for the symmetric system where ε = 0, the population decay predicted by MASH is unaffected by application of the decoherence correction, leaving it in perfect agreement with Marcus theory. In contrast, however, the FSSH results are made significantly worse by application of the decoherence correction, for reasons explained below. For the asymmetric system, *βε* = 3, we see that application of the decoherence correction improves the original MASH result, removing the ∼40% error and bringing it into almost perfect agreement with Marcus theory. Again, however, the simple decoherence correction does not fix FSSH, in this case taking the rate from being too fast to too slow. These results can be understood by noting that by applying the decoherence correction far from the crossing region we simply make the long-time dynamics consistent with the short-time dynamics. For MASH, the short-time dynamics has the correct rate, but for FSSH, the short-time dynamics is wrong, as one can see from the inset, and hence, we recover the spuriously low rate shown in Figure 1.

That such simple decoherence corrections do not fix FSSH is not a new observation, and for this reason, many far more sophisticated decoherence approaches have been developed.^28,29^ However, these methods often come with additional disadvantages, such as increased cost, and as they are ad hoc, they are not always guaranteed to improve the results. The point we stress here is that the increased accuracy of MASH at short times means that decoherence corrections can be applied much more infrequently. For many ultrafast problems, this means that they may not be needed at all, but when they are needed, they can be both safer and simpler.

**Figure 5:**
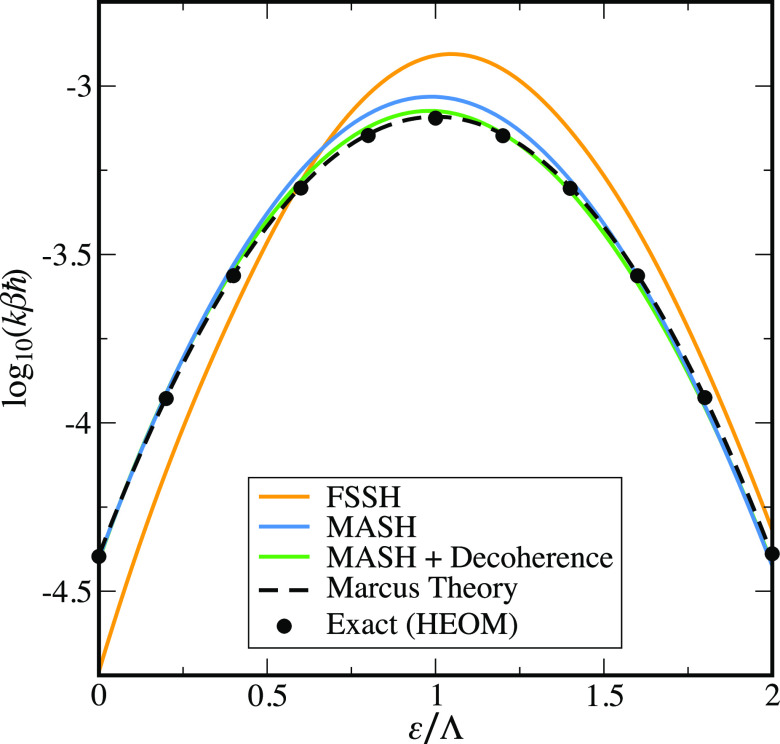
Logarithmic plot of the rate vs reaction driving force, showing the famous Marcus turnover behavior for a spin-boson model with weak diabatic coupling, log_10_(*βΔ*) = −^7^/_5_, βℏΩ = ^1^/_4_, γ = Ω, and *βΛ* = 12. FSSH and MASH rates were calculated from the slope of ⟨*P*_p_(*t*)⟩, at the plateau time, between *t* = 10βℏ and *t* = 20βℏ.

Finally, having understood the difference between MASH and FSSH, we consider the famous Marcus turnover curve. Figure 5 shows the behavior of the rate in the weak-coupling limit [log_10_(*βΔ*) = −^7^/_5_] as a function of the bias to products, ε. As in Figure 1, the FSSH and MASH rates are calculated from the slope of ⟨*P*_p_(*t*)⟩ near the plateau time, between *t* = 10βℏ and *t* = 20βℏ. As expected from the results presented above, FSSH deviates significantly from Marcus theory and the exact results, showing an unphysical asymmetry around ε = Λ. In contrast, MASH reproduces both the exact results and Marcus theory very well. MASH is also not perfectly symmetric due to a slightly larger error deep in the inverted regime (ε = 2Λ) than in the symmetric case (ε = 0). However, in both cases, the errors are <10%. The largest error in MASH is observed close to the activationless limit ε/Λ = 1. Here the MASH rate is ∼15% higher than the Marcus theory result. In contrast, FSSH is ∼60% too large. As the avoided crossing is located at the minimum of the reactant well in the activationless case, this leads to a faster buildup of overcoherence error and the increase in the rate seen at long times in the bottom panel of Figure 4 starts to affect the dynamics even at the short times considered here. This is confirmed by application of the same decoherence correction that was used in Figure 4, which stops the buildup of overcoherence error, resulting in MASH rates that are within 7% of the exact rate for the full range of ε considered.^f^ As in Figure 4, the increased error of FSSH at short times means that this simple decoherence correction is not sufficient to fix the inconsistency error of FSSH, and hence, the rates still deviate significantly from the Marcus theory result, as one can see in Figure S6.

In conclusion, it is well established that the overcoherence error of FSSH is most pronounced for the calculation of reaction rates in the limit of weak diabatic coupling (the Marcus theory regime).^31−35^ Here we revisit this problem to assess the accuracy of a newly proposed alternative to FSSH, the mapping approach to surface hopping (MASH). In comparing MASH and FSSH, we have considered two different time scales: the time scale of a single barrier crossing event, *t*_pl_, and the time scale of the reaction, τ_rxn_.

On the time scale of barrier crossing, MASH provides a significant improvement upon FSSH, accurately recovering the results of Marcus theory without the use of decoherence corrections. This might seem surprising at first as it is not immediately obvious how MASH, which is also an independent trajectory method, can capture decoherence. However, we have shown that the improvement can be explained in terms of the dramatic inconsistency between the active state and wave function coefficients, which can exist in FSSH but is absent from MASH.

On very long time scales, MASH again provides a significant improvement over FSSH. While overcoherence error does still build up in MASH, we have found it to be much less significant than in FSSH. This can again be explained in terms of the inconsistency in FSSH, which means that the buildup of error can be sudden and large, whereas in MASH the buildup of error is more gradual and ultimately smaller. Perhaps most importantly, the increased accuracy of MASH compared to that of FSSH at short times means that when they are used, decoherence corrections need only be applied well away from the coupling region, making them safer and simpler to use.

MASH also has additional practical advantages over FSSH in the calculation of reaction rates. In particular, as the dynamics of MASH are deterministic and obey time-translation symmetry, there is no need for approximate backward-time propagation or advanced methods such as forward-flux sampling. One can rigorously apply the flux-correlation formalism and related techniques, such as the Bennett–Chandler method, to efficiently calculate reaction rates. Given these significant improvements and the fact that MASH is simple to use, requires only relatively minor modifications to existing FSSH code, and can be run at equivalent computational cost, MASH has the potential to replace FSSH as the go-to method for the simulation of nonadiabatic processes.

The only thing limiting MASH as a replacement for FSSH is that the current theory is restricted to two-state problems. Recently, a modification to MASH has been proposed, designed for application to multistate problems.^70^ However, this theory is different from the MASH described here. It does not reduce to the current theory in the case of a two-level system, and although it is accurate for many problems, it was shown to be significantly less accurate for the time scales of population decay in a spin-boson model in the inverted regime. Work to develop a multistate generalization of the present MASH method is in progress, and if this can be achieved, while retaining the advantages of the two-state theory, it would present a significant challenge to the hegemony of FSSH.

Finally, we note that we have focused exclusively on the limit of classical nuclei. It is, however, well-known that nuclear quantum effects, in particular tunneling and zero-point energy, can have a significant effect on the rate of nonadiabatic reactions, such as electron transfer, intersystem crossing, and proton-coupled electron transfer.^71−78^ In recent years, there has been a continued interest in the development of methods that can accurately incorporate nuclear quantum effects into the simulation of electronically nonadiabatic reactions.^79−85^ While there has been significant development in methods specialized for accurately predicting thermal reaction rates,^86−91^ at present there is no fully dynamical method that can offer comparable accuracy.^92^ This is in part due to the difficulty that such dynamical methods face in accurately describing rates, even in the limit of classical nuclei. In this regard, the results of this study indicate that MASH provides a new and exciting route to the development of a fully dynamic nonadiabatic theory capable of accurately describing nuclear tunneling and zero-point energy.

## Supporting Information Available

Additional methodological details and figures illustrating the equivalence of different dividing surfaces, the MASH flux-correlation formalism, rates as a function of Δ for asymmetric systems, rates as a function of Δ for an overdamped system, behavior of FSSH Marcus turnover with decoherence, results for a system with a large reorganization energy, and equivalence of MASH and MASH+decoherence results in the prediction of Δ^2^ behavior (PDF)

## Footnotes

a See the Supporting Information for full mathematical details.

b This is because the parts of the thermal distribution corresponding to reactants and products are two regions of high probability density that are well separated in phase space. Hence, any definition that respects this separation will give the same long-time dynamics. This is true regardless of whether MASH and FSSH obey detailed balance globally provided they do so in the reactant and product wells, which is trivially the case when the system is electronically adiabatic in these regions.

c See also Figure S4.

d It is interesting to note that, despite FSSH and MASH predicting incorrect rates at long times, they nevertheless approach the correct equilibrium populations. This has been observed in previous studies of FSSH,^68^ and for MASH, it was recently proven that under the assumption of ergodicity it is guaranteed to approach the correct long time limit.^69^

e In this system, this is equivalent to using the more common derivative coupling-based cutoff of ref (24). We choose to give the equivalent energy gap for ease of interpretability. Additionally, we note that the results in Figure 4 are unchanged if the decoherence correction is applied at every step where *V*_+_ – *V*_–_ > 4*k*_B_*T* or only when the energy gap reaches a local maximum along the trajectory greater than the threshold. The latter was the actual implementation used for the figures.

f Note that, close to the activationless regime, ε ≈ Λ, where the crossing occurs in the reactant well, the results are somewhat sensitive to how the decoherence correction is applied and the exact value of the cutoff used. We wish to stress that this is not intended as a serious suggestion for a universal decoherence scheme but is used instead to illustrate the key points made in the text.
