# Supplementary material for: Recovering Marcus Theory Rates and Beyond without the Need for Decoherence Corrections: The Mapping Approach to Surface Hopping
Source: J Phys Chem Lett. 2024 Jan 12;15(3):707–16. doi: 10.1021/acs.jpclett.3c03197 (PMC10823533; doi:10.1021/acs.jpclett.3c03197)
Supplement: Supplementary file 1 — jz3c03197_si_001.pdf [file jz3c03197_si_001.pdf]

Supporting Information: Recovering Marcus Theory Rates and Beyond without the Need for Decoherence Corrections: The Mapping Approach to Surface Hopping

Joseph E. Lawrence,[*,†] Jonathan R. Mannouch,[*,‡] and Jeremy O. Richardson[*,†]

†*Department of Chemistry and Applied Biosciences, ETH Zurich, 8093 Zurich, Switzerland*

‡*Hamburg Center for Ultrafast Imaging, Universität Hamburg and the Max Planck Institute for the Structure and Dynamics of Matter, Luruper Chaussee 149, 22761 Hamburg, Germany*

E-mail: joseph.lawrence@phys.chem.ethz.ch; jonathan.mannouch@mpsd.mpg.de; jeremy.richardson@phys.chem.ethz.ch

Key Definitions.

To simplify notation, it is helpful to define the trace (or phase-space integral) over the MASH variables as

$$\mathrm{tr}[A(\boldsymbol{p},\boldsymbol{q},\boldsymbol{S})] = \frac{1}{(2\pi\hbar)^f}\int \mathrm{d}\boldsymbol{q}\int \mathrm{d}\boldsymbol{p}\int \mathrm{d}\boldsymbol{S}A(\boldsymbol{p},\boldsymbol{q},\boldsymbol{S}), \tag{S1}$$

where f is the number of nuclear degrees of freedom. The integral over $\boldsymbol{S}$ is defined as

$$\int \mathrm{d}\boldsymbol{S}A(\boldsymbol{S}) = \frac{1}{2\pi}\int_0^\pi \mathrm{d}\theta\int_0^{2\pi}\mathrm{d}\phi\sin(\theta)A(\boldsymbol{S}), \tag{S2}$$

with

$$S_x = \sin(\theta)\cos(\phi) \tag{S3a}$$

$$S_y = \sin(\theta)\sin(\phi) \tag{S3b}$$

$$S_z = \cos(\theta). \tag{S3c}$$

The trace over the FSSH variables can be defined similarly, the key difference being that, as the active surface, n, is not obtained deterministically from the Bloch sphere, it must be introduced as a separate variable. Hence, we define the FSSH trace as

$$\mathrm{tr}[A(\boldsymbol{p}, \boldsymbol{q}, \boldsymbol{S}, n)] = \frac{1}{(2\pi\hbar)^f} \sum_{n=\pm} \int \mathrm{d}\boldsymbol{q} \int \mathrm{d}\boldsymbol{p} \int \mathrm{d}\boldsymbol{S} A(\boldsymbol{p}, \boldsymbol{q}, \boldsymbol{S}, n). \tag{S4}$$

It will also be helpful to define the MASH and FSSH total energy as

$$E(\boldsymbol{p}, \boldsymbol{q}, n) = H_+(\boldsymbol{p}, \boldsymbol{q})\delta_{+,n} + H_-(\boldsymbol{p}, \boldsymbol{q})\delta_{-,n}, \tag{S5}$$

where

$$H_\pm(\boldsymbol{p}, \boldsymbol{q}) = \sum_{j=1}^{f} \frac{p_j^2}{2m_j} + V_\pm(\boldsymbol{q}), \tag{S6}$$

are the energies on each of the adiabatic states, and we remind the reader that in MASH

$$n(\boldsymbol{S}) = \mathrm{sign}(S_z). \tag{S7}$$

Sampling of the Bloch Sphere.

For a system that is initially in a pure adiabatic state, the MASH derivation[S1] leads to a weight factor proportional to $|S_z|$ in the calculation of expectation values. In the present work we incorporate this weight factor into the initial sampling of the vector on the Bloch-Sphere. This can be achieved by noting that for a system initially on adiabatic state, a, we have

$$\begin{aligned}
&\frac{\mathrm{tr}\big[A(\boldsymbol{p}, \boldsymbol{q})\delta_{a,n(\boldsymbol{S})}|S_z|B(\boldsymbol{p}(t), \boldsymbol{q}(t), \boldsymbol{S}(t))\big]}{\mathrm{tr}\big[A(\boldsymbol{p}, \boldsymbol{q})\delta_{a,n(\boldsymbol{S})}|S_z|\big]} \\
=&\frac{\int \mathrm{d}\boldsymbol{q} \int \mathrm{d}\boldsymbol{p} \int \mathrm{d}\boldsymbol{S}\, A(\boldsymbol{p}, \boldsymbol{q})|S_z|\delta_{a,n(\boldsymbol{S})}B(\boldsymbol{p}(t), \boldsymbol{q}(t), \boldsymbol{S}(t))}{\int \mathrm{d}\boldsymbol{q} \int \mathrm{d}\boldsymbol{p} \int \mathrm{d}\boldsymbol{S}\, A(\boldsymbol{p}, \boldsymbol{q})|S_z|\delta_{a,n(\boldsymbol{S})}} \\
=&\int \mathrm{d}\boldsymbol{q} \int \mathrm{d}\boldsymbol{p} \int \mathrm{d}\boldsymbol{S}\, \rho_A(\boldsymbol{p}, \boldsymbol{q})\rho_a(\boldsymbol{S})B(\boldsymbol{p}(t), \boldsymbol{q}(t), \boldsymbol{S}(t))
\end{aligned} \tag{S8}$$

where $\rho_a(\boldsymbol{S})$ is the normalised probability density function used to sample the Bloch sphere for a system initially on state a

$$\rho_a(\boldsymbol{S}) = \frac{|S_z|\delta_{a,n(\boldsymbol{S})}}{\int \mathrm{d}\boldsymbol{S}|S_z|\delta_{a,n(\boldsymbol{S})}}. \tag{S9}$$

Note this is equivalent to sampling θ and ϕ from the distribution

$$\rho_a(\theta,\phi) = \frac{\sin(\theta)\,|\cos(\theta)|\,h(a\cos(\theta))}{\int_0^\pi \mathrm{d}\theta \int_0^{2\pi} \mathrm{d}\phi\, \sin(\theta)\,|\cos(\theta)|\,h(a\cos(\theta))}, \tag{S10}$$

which is done by uniformly sampling $u \in [0,1)$ and $v \in (0,1]$ and setting

$$\phi = 2\pi u \tag{S11a}$$

$$\theta = \operatorname{acos}\big(a\sqrt{v}\big). \tag{S11b}$$

Decoherence corrections.

Ref. S1 shows how the application of 'quantum jumps' allows one to systematically converge MASH towards the QCLE. Furthermore, it was shown that in cases where $\langle S_x\rangle = \langle S_y\rangle = 0$, the quantum jump can be simplified to give a rigorous decoherence correction. Just as with the initial sampling described above, the resampling of the spin-vector and weight factor described in Ref. S1, can be combined. This is what is done in the present work. For a system on active surface $n(\boldsymbol{S}) = a$ the decoherence correction is performed by just resampling the $\boldsymbol{S}$ vector from the initial distribution given above, $\rho_a(\boldsymbol{S})$.

Numerical calculation of MASH and FSSH rates.

For a system which undergoes incoherent dynamics at long time, the rate constant to pass from reactants to products can be written formally as[S2,S3]

$$k = \lim_{t\to\infty} \frac{\frac{\mathrm{d}}{\mathrm{d}t}\langle P_p(t)\rangle}{1 - \langle P_p(t)\rangle/\langle P_p(\infty)\rangle}. \tag{S12}$$

Often this is simplified by introducing the idea of the plateau time, $t_{\rm pl}$,[S4] which is short enough that $\langle P_p(t)\rangle/\langle P_p(\infty)\rangle \approx 0$, but long enough that the dynamics has settled into the exponential

decay, such that

$$k \approx \frac{\mathrm{d}}{\mathrm{d}t}\langle P_p(t)\rangle\bigg|_{t=t_{\mathrm{pl}}}. \tag{S13}$$

For the results presented in the main paper, we compute the MASH and FSSH rates by taking the average of

$$k(t) = \frac{\frac{\mathrm{d}}{\mathrm{d}t}\langle P_p(t)\rangle}{1 - \langle P_p(t)\rangle/\langle P_p(\infty)\rangle} \tag{S14}$$

between $t = 10\beta\hbar$ and $t = 20\beta\hbar$, where the derivative is computed by finite difference from $\langle P_p(t)\rangle$. In MASH this is defined as

$$\langle P_p(t)\rangle = \frac{\mathrm{tr}\left[e^{-\beta E}|S_z|P_r P_p(t)\right]}{\mathrm{tr}\left[e^{-\beta E}|S_z|P_r\right]}, \tag{S15}$$

and in FSSH as

$$\langle P_p(t)\rangle = \frac{\mathrm{tr}\left[e^{-\beta E}\delta(S_z - n)P_r P_p(t)\right]}{\mathrm{tr}\left[e^{-\beta E}\delta(S_z - n)P_r\right]}. \tag{S16}$$

As stated in the body of the main paper, in both MASH and FSSH we use the following general definition of the reactants and products, valid in both the normal and inverted Marcus regimes,

$$P_p^{(g)}(t) = h(U_0(t) - U_1(t))\delta_{n(t),-} + h(U_1(t) - U_0(t))\delta_{n(t),+} \tag{S17a}$$

$$P_r^{(g)}(t) = h(U_0(t) - U_1(t))\delta_{n(t),+} + h(U_1(t) - U_0(t))\delta_{n(t),-}, \tag{S17b}$$

where $h(x)$ is the Heaviside step function. Here we have introduced an additional superscript (g) to distinguish this general definition from the position, and adiabatic definitions that are not used in the main paper, but are considered in this supporting information. We remind the reader that $P_r^{(g)} = 1 - P_p^{(g)}$.

Equivalence of different definitions of reactants and products.

Here we demonstrate numerically that the definition of reactants and products given in Eq. S17 give rates which are identical to more common definitions of reactants and products. Firstly in the normal regime, we compare to a purely position space definition of the reactants and products

$$P_p^{(q)}(t) = h(U_0(t) - U_1(t)) \tag{S18a}$$

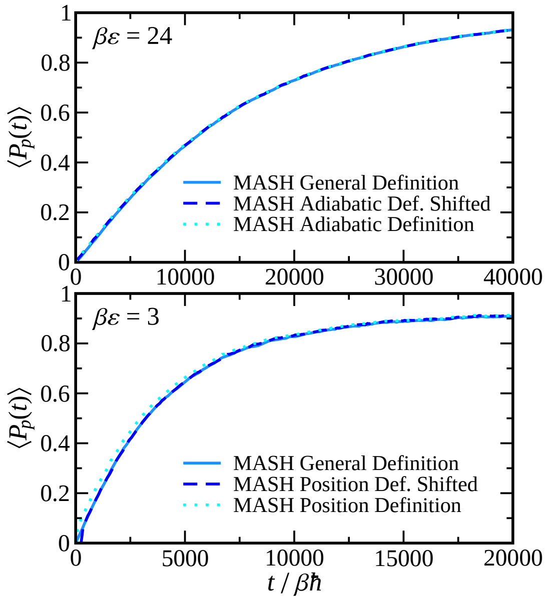

Figure S1: Comparison of $\langle P_p(t)\rangle$ for different definitions of the reactants and products at long time. Upper panel: inverted regime $\beta\varepsilon = 24$, with $\log_{10}(\beta\Delta) = -7/5$, $\beta\hbar\Omega = 1/4$, $\gamma = \Omega$ and $\beta\Lambda = 12$. The dashed curve is shifted by $t = 60\beta\hbar$ to better illustrate the equivalence with the general definition at long time. Lower panel: normal regime $\beta\varepsilon = 3$, with $\log_{10}(\beta\Delta) = -7/5$, $\beta\hbar\Omega = 1/4$, $\gamma = \Omega$ and $\beta\Lambda = 12$. The dashed curve is shifted by $t = 250\beta\hbar$ to better illustrate the equivalence with the general definition at long time.

$$P_r^{(q)}(t) = h(U_1(t) - U_0(t)). \tag{S18b}$$

Secondly, in the inverted regime we compare to using a purely adiabatic state definition of the reactants and products

$$P_p^{(a)}(t) = \delta_{n(t),-} \tag{S19a}$$

$$P_r^{(a)}(t) = \delta_{n(t),+}. \tag{S19b}$$

Figure S1 compares $\langle P_p(t)\rangle$ calculated using the general definition (used in the main paper) against the position and adiabatic definitions. The lower panel shows the results for a system in the normal regime and the upper panel shows the results for a system in the inverted regime. We see in both cases that at long time the definition used in the main paper is equivalent to the more commonly used definitions given in Eqs. S18 and S19.

Flux-Correlation version of MASH.

It is typically more efficient to calculate $\frac{\mathrm{d}}{\mathrm{d}t}\langle P_p(t)\rangle$ using the flux-correlation formalism, rather than from a direct simulation of $\langle P_p(t)\rangle$.[S4] To arrive at the flux-correlation formalism, one differentiates analytically and then shifts the time origin. As discussed in the main paper, it is not possible to do this within FSSH. However it is possible for MASH. The following demonstrates that this is the case and derives the resulting flux-correlation MASH expressions.

Time symmetries.

MASH dynamics obeys a number of important properties that allow one to use the flux-correlation formalism to efficiently calculate reaction rates.

Time translation.

The first of these properties is time-translation symmetry. As the dynamics has no explicit time dependence, we can begin by simply changing the origin of time to give

$$\begin{aligned}\mathrm{tr}\Big[e^{-\beta E(\boldsymbol{p}_0,\boldsymbol{q}_0,\boldsymbol{S}_0)}A(0)B(t)\Big] &= \frac{1}{(2\pi\hbar)^f}\int \mathrm{d}\boldsymbol{q}_0\int \mathrm{d}\boldsymbol{p}_0\int \mathrm{d}\boldsymbol{S}_0\, e^{-\beta E(\boldsymbol{p}_0,\boldsymbol{q}_0,\boldsymbol{S}_0)}A(0)B(t)\\ &= \frac{1}{(2\pi\hbar)^f}\int \mathrm{d}\boldsymbol{q}_{-t}\int \mathrm{d}\boldsymbol{p}_{-t}\int \mathrm{d}\boldsymbol{S}_{-t}\, e^{-\beta E(\boldsymbol{p}_{-t},\boldsymbol{q}_{-t},\boldsymbol{S}_{-t})}A(-t)B(0).\end{aligned} \tag{S20}$$

Now as proved in the original MASH paper (Ref. S1) the MASH dynamics conserve both the measure and the energy, hence

$$\begin{aligned}\mathrm{tr}\Big[e^{-\beta E(\boldsymbol{p}_0,\boldsymbol{q}_0,\boldsymbol{S}_0)}A(0)B(t)\Big] &= \frac{1}{(2\pi\hbar)^f}\int \mathrm{d}\boldsymbol{q}_0\int \mathrm{d}\boldsymbol{p}_0\int \mathrm{d}\boldsymbol{S}_0\, e^{-\beta E(\boldsymbol{p}_0,\boldsymbol{q}_0,\boldsymbol{S}_0)}A(-t)B(0)\\ &= \mathrm{tr}\Big[e^{-\beta E(\boldsymbol{p}_0,\boldsymbol{q}_0,\boldsymbol{S}_0)}A(-t)B(0)\Big].\end{aligned} \tag{S21}$$

It is important to note that, as discussed in the original MASH paper,[S1] MASH does not exactly obey detailed balance for the calculation of reaction rates. This is because the dynamics does not in general conserve $|S_z(t)|$ and hence

$$\mathrm{tr}\left[e^{-\beta E}|S_z|P_rP_p(t)\right] \neq \mathrm{tr}\left[e^{-\beta E}|S_z|P_pP_r(-t)\right]. \tag{S22}$$

Therefore while the dynamics does obey time-translational symmetry, the correlation functions do not when $|S_z|$ is treated as part of the initial distribution. However, as we shall explain below, the fact that the dynamics obey time-translation symmetry is enough to develop an efficient flux-correlation formalism. Finally we note that although MASH does not formally obey detailed balance, under the assumption of ergodicity, MASH is guaranteed to correctly thermalise in the long-time limit.[S5] Hence MASH is accurate both at short time and at long time.

Time-inversion symmetry.

The MASH equations of motion are given, in the adiabatic basis, by

$$\dot{S}_x = \sum_j \frac{2d_j(\boldsymbol{q})p_j}{m_j} S_z - \frac{V_+(\boldsymbol{q}) - V_-(\boldsymbol{q})}{\hbar} S_y \tag{S23a}$$

$$\dot{S}_y = \frac{V_+(\boldsymbol{q}) - V_-(\boldsymbol{q})}{\hbar} S_x \tag{S23b}$$

$$\dot{S}_z = -\sum_j \frac{2d_j(\boldsymbol{q})p_j}{m_j} S_x \tag{S23c}$$

$$\dot{q}_j = \frac{p_j}{m_j} \tag{S23d}$$

$$\dot{p}_j = -\frac{\partial V_+(\boldsymbol{q})}{\partial q_j} h(S_z) - \frac{\partial V_-(\boldsymbol{q})}{\partial q_j} h(-S_z) + 2[V_+(\boldsymbol{q}) - V_-(\boldsymbol{q})] d_j(\boldsymbol{q}) S_x \delta(S_z). \tag{S23e}$$

These equations of motion are left unchanged under the transformation: $t \mapsto -t$, $S_y \mapsto -S_y$ and $p_j \mapsto -p_j$. This means than any expression involving backward-propagated trajectories can be converted into an expression involving forward-propagated trajectories, by reversing the signs of S_y and p_j.

Flux-correlation function.

Using the time-translation symmetry, we can straightforwardly obtain the following general expression for the product population derivative

$$\frac{\mathrm{d}}{\mathrm{d}t}\langle P_p(t)\rangle = \frac{\mathrm{tr}\left[e^{-\beta E}\dot{P}_p(0)P_r(-t)|S_z(-t)|\right]}{\mathrm{tr}\left[e^{-\beta E}P_r|S_z|\right]}. \tag{S24}$$

Before we can use the time-inversion symmetry to simplify further, we need to specialise to the particular definition of the reactants and products. In the following, we consider first the MASH

flux-correlation formalism when the reactants and products are defined in terms of position or adiabatic states, as in Eqs. S18 and S19. Then we will consider the flux-correlation formalism for the general definition of reactants and products used in the main paper (Eq. S17), which combines results from both the position and adiabatic definitions. In each case, we need to evaluate the derivative, $\dot{P}_p(0)$.

Position based definition (normal regime).

We begin with considering a position based definition. To do so, it is helpful to simplify notation by defining the generalised coordinate, Q, such that $Q = Q^\ddagger$ corresponds to $U_0(\boldsymbol{q}) = U_1(\boldsymbol{q})$. With this definition, we have that

$$P_r^{(q)}(t) = h(-(Q(t) - Q^\ddagger)) \tag{S25a}$$

$$P_p^{(q)}(t) = h(Q(t) - Q^\ddagger). \tag{S25b}$$

Hence, evaluating the time derivative of $P_p^{(q)}(t)$, we obtain

$$\dot{P}_p^{(q)}(t) = \dot{Q}(t)\delta(Q(t) - Q^\ddagger). \tag{S26}$$

Inserting this into Eq. S24 and employing the time-inversion symmetry, we obtain

$$\langle \dot{P}_p^{(q)}(t) \rangle = - \frac{\mathrm{tr}\left[e^{-\beta E} \dot{Q} \delta(Q - Q^\ddagger) |S_z(t)| P_r^{(q)}(t) \right]}{\mathrm{tr}\left[e^{-\beta E} P_r^{(q)} |S_z| \right]}. \tag{S27}$$

Except for the MASH weighting factor, $|S_z(t)|$, this has an identical form to the standard approach for calculating classical rates within the Born–Oppenheimer approximation.[S4] We note that by considering the equivalent expression calculated with the MASH weighting factor at the initial time, $|S_z(0)|$, one obtains a very useful estimate of the MASH error.[S1]

Adiabatic definition (inverted regime).

To evaluate the derivative of $P_p(t)$ in Eq. S19, it is helpful to first use the definition of the active state in MASH to rewrite the reactant and products as

$$P_r^{(a)}(t) = h(S_z(t)) \tag{S28a}$$

$$P_p^{(a)}(t) = h(-S_z(t)) = 1 - h(S_z(t)). \tag{S28b}$$

Evaluating the derivative requires care, as the MASH dynamics is not analytic at t_{hop} (where $S_z(t_{\text{hop}}) = 0$) and hence

$$\frac{\mathrm{d}}{\mathrm{d}t} h(S_z(t)) \neq \dot{S}_z(t)\delta(S_z(t)). \tag{S29}$$

We therefore return to the definition of the derivative

$$\frac{\mathrm{d}}{\mathrm{d}t} h(S_z(t)) = \lim_{\epsilon\to 0} \frac{h(S_z(t+\epsilon)) - h(S_z(t))}{\epsilon}, \tag{S30}$$

and carefully take the limit such that t is not exactly the hopping time, but rather (for $\epsilon \to 0^+$) infinitesimally before or (for $\epsilon \to 0^-$) infinitesimally after the hop. To evaluate the derivative we thus need to consider the behaviour of $h(S_z(t+\epsilon)) - h(S_z(t))$ for small values of ϵ. It is clear that this can only be non-zero if there is a change in state between the time t and $t+\epsilon$. Assuming that ϵ is small enough that there is at most one attempted hop (where $S_z = 0$) between t and $t+\epsilon$, a careful consideration of the possibilities (explained in detail below) results in the equation

$$h(S_z(t+\epsilon)) - h(S_z(t)) = \Big[h(S_z(t) + \epsilon\dot{S}_z(t)) - h(S_z(t))\Big]\Big[h(S_z(t)) + h(-S_z(t))\, h(\mathit{\Delta} E_{\text{hop}}(t))\Big], \tag{S31}$$

where $\mathit{\Delta} E_{\text{hop}}$ is the difference between the kinetic energy along the derivative coupling vector and the adiabatic energy gap

$$\mathit{\Delta} E_{\text{hop}} = \frac{1}{2}\frac{(\tilde{\boldsymbol{p}}\cdot\tilde{\boldsymbol{d}})^2}{\tilde{\boldsymbol{d}}\cdot\tilde{\boldsymbol{d}}} - (V_+(\boldsymbol{q}) - V_-(\boldsymbol{q})), \tag{S32}$$

where $\tilde{p}_j = p_j/\sqrt{m_j}$ and $\tilde{d}_j = d_j/\sqrt{m_j}$ are the mass-weighted momentum and derivative coupling vectors respectively. To understand Eq. S31, we note first that for the left-hand side to be non-zero the active state must change between t and $t+\epsilon$. The first of the two terms on the right-hand side

of Eq. S31 corresponds to whether a hop is attempted and the second to whether it has enough energy to actually occur. The first term is only non-zero if the current rate of change predicts a change in the sign of S_z between t and $t+\epsilon$ (i.e. an attempted hop). The second term then accounts for the possibility that the hop is frustrated, in which case the overall expression must be zero (due to the reflection of S_z off the spin-sphere equator for a frustrated hop). To see that this is the correct expression, note that if the system is on the upper surface, $h(S_z(t)) = 1$, then the hop is necessarily not frustrated (this is true whether t is just before or just after a hop, i.e. whether ϵ is positive or negative). However, if the system is on the lower surface, $h(-S_z(t)) = 1$, then it will hop (or just have hopped) if and only if the kinetic energy along the derivative coupling vector is greater than the adiabatic energy gap, $\mathit{\Delta} E_{\text{hop}}(t) > 0$.

As we have already alluded to, there is a choice in whether the limit is taken from above ($\epsilon \to 0^+$) or from below ($\epsilon \to 0^-$). First we will take the limit as $\epsilon \to 0^-$. This will result in expressions for which t is infinitesimally after the hopping time, rather than before. Noting that only the first term on the right-hand side of Eq. S31 involves ϵ, the key to evaluating the limit is the following identity

$$\begin{aligned}\lim_{\epsilon\to 0^-} \frac{h(x+\epsilon b)-h(x)}{\epsilon} &= \lim_{\eta\to 0^+} \delta(x-\eta)h(b)b + \lim_{\eta\to 0^-} \delta(x-\eta)h(-b)b \\ &= \delta_+(x)h(b)b + \delta_-(x)h(-b)b,\end{aligned} \tag{S33}$$

where we will replace x and b with $S_z(t)$ and $\dot{S}_z(t)$. This result is straightforward to verify by considering the two cases $b > 0$ and $b < 0$ separately. Combining this with Eq. S31, and utilising trivial identities such as $\delta_+(S_z(t))h(-S_z(t)) = 0$, one then obtains the following expression for the time derivative

$$\frac{\mathrm{d}}{\mathrm{d}t} h(S_z(t)) = \delta_+(S_z(t))\, h(\dot{S}_z(t))\dot{S}_z(t) + \delta_-(S_z(t))\, h(-\dot{S}_z(t))\dot{S}_z(t)\, h(\mathit{\Delta} E_{\text{hop}}(t)). \tag{S34}$$

Physically these two terms correspond to trajectories that have $S_z(t)$ infinitesimally greater than zero for which $S_z(t)$ is increasing (i.e. trajectories that have just hopped up), and trajectories with enough energy to hop that have $S_z(t)$ infinitesimally less than zero for which $S_z(t)$ is decreasing (i.e. trajectories which have just hopped down).

Following the same line of reasoning it can be shown that taking the limit ($\epsilon \to 0^+$) leads instead to

$$\frac{\mathrm{d}}{\mathrm{d}t} h(S_z(t)) = \delta_+(S_z(t))h(-\dot{S}_z(t))\dot{S}_z(t) + \delta_-(S_z(t))h(\dot{S}_z(t))\dot{S}_z(t)h(\mathit{\Delta} E_{\text{hop}}(t)). \tag{S35}$$

One can therefore equally weight these two expressions to obtain the symmetric expression

$$\frac{\mathrm{d}}{\mathrm{d}t} h(S_z(t)) = \frac{\dot{S}_z(t)}{2}[\delta_+(S_z(t)) + \delta_-(S_z(t))h\left(\mathit{\Delta} E_{\text{hop}}(t)\right)]. \tag{S36}$$

Each of these three expressions (Eqs. S34, S35 and S36) are equally valid, although they may be more or less practical. Considering first the symmetric definition, we see that inserting Eq. S36 into Eq. S24 and making use of the time-inversion symmetry gives

$$\begin{aligned} \frac{\mathrm{d}}{\mathrm{d}t}\langle P_p^{(a)}(t)\rangle =& \frac{1}{2}\frac{\mathrm{tr}\left[e^{-\beta E}\delta_+(S_z)\dot{S}_z h(S_z(t))|S_z(t)|\right]}{\mathrm{tr}\left[e^{-\beta E}h(S_z)|S_z|\right]} \\ &+\frac{1}{2}\frac{\mathrm{tr}\left[e^{-\beta E}\delta_-(S_z)\dot{S}_z h\left(\mathit{\Delta} E_{\text{hop}}\right)h(S_z(t))|S_z(t)|\right]}{\mathrm{tr}\left[e^{-\beta E}h(S_z)|S_z|\right]}. \end{aligned} \tag{S37}$$

This expression has the advantage that it is symmetric, although numerical implementation will involve trajectories that hop during the first time step, which one might want to avoid. Alternatively, therefore, one can make use of Eq. S35, which has the hop infinitesimally after t. This might seem like it is the wrong choice, but after inserting into Eq. S24 and making use of the time-inversion symmetry the hop will be infinitesimally before $t = 0$, resulting in the following expression

$$\begin{aligned} \frac{\mathrm{d}}{\mathrm{d}t}\langle P_p^{(a)}(t)\rangle =& \frac{\mathrm{tr}\left[e^{-\beta E}\delta_+(S_z)h(\dot{S}_z)\dot{S}_z h(S_z(t))|S_z(t)|\right]}{\mathrm{tr}\left[e^{-\beta E}h(S_z)|S_z|\right]} \\ &+\frac{\mathrm{tr}\left[e^{-\beta E}\delta_-(S_z)h(-\dot{S}_z)\dot{S}_z h\left(\mathit{\Delta} E_{\text{hop}}\right)h(S_z(t))|S_z(t)|\right]}{\mathrm{tr}\left[e^{-\beta E}h(S_z)|S_z|\right]}. \end{aligned} \tag{S38}$$

General definition (used in main paper).

Combining the results from the previous two sections we see that the general definition of reactants and products used in the main paper (Eqs. S17 and **??**) can be equivalently written as

$$P_r^{(g)} = h(-(Q - Q^{\ddagger}))h(-S_z) + h(Q - Q^{\ddagger})h(S_z) \tag{S39}$$

$$P_p^{(g)} = h(Q - Q^{\ddagger})h(-S_z) + h(-(Q - Q^{\ddagger}))h(S_z). \tag{S40}$$

Taking the derivative with respect to time, making use of Eqs. S26 and S36, gives

$$\begin{aligned} \dot{P}_p^{(g)} &= \dot{Q}\delta(Q - Q^{\ddagger})[h(-S_z) - h(S_z)] \\ &+ \frac{1}{2}[h(-(Q - Q^{\ddagger})) - h(Q - Q^{\ddagger})][\delta_+(S_z)\dot{S}_z + \delta_-(S_z(t))\dot{S}_z h(\mathit{\Delta} E_{\text{hop}})]. \end{aligned} \tag{S41}$$

Inserting this into Eq. S24 and making use of the time-inversion symmetry we obtain

$$\begin{aligned} \frac{\mathrm{d}}{\mathrm{d}t}\langle P_p^{(g)}(t)\rangle =& \frac{\operatorname{tr}\left[e^{-\beta E} P_r^{(g)}(t)|S_z(t)|\dot{Q}\delta(Q - Q^{\ddagger})[h(S_z) - h(-S_z)]\right]}{\operatorname{tr}\left[e^{-\beta E}[h(-(Q - Q^{\ddagger}))h(-S_z) + h(Q - Q^{\ddagger})h(S_z)]|S_z|\right]} \\ &+ \frac{\operatorname{tr}\left[e^{-\beta E} P_r^{(g)}(t)|S_z(t)|[h(Q - Q^{\ddagger}) - h(-(Q - Q^{\ddagger}))]\delta_+(S_z)\dot{S}_z\right]}{2\operatorname{tr}\left[e^{-\beta E}[h(-(Q - Q^{\ddagger}))h(-S_z) + h(Q - Q^{\ddagger})h(S_z)]|S_z|\right]} \\ &+ \frac{\operatorname{tr}\left[e^{-\beta E} P_r^{(g)}(t)|S_z(t)|[h(Q - Q^{\ddagger}) - h(-(Q - Q^{\ddagger}))]\delta_-(S_z)\dot{S}_z h\left(\mathit{\Delta} E_{\text{hop}}\right)\right]}{2\operatorname{tr}\left[e^{-\beta E}[h(-(Q - Q^{\ddagger}))h(-S_z) + h(Q - Q^{\ddagger})h(S_z)]|S_z|\right]}. \end{aligned} \tag{S42}$$

Here we have chosen to use the symmetric definition of the derivative of $h(S_z(t))$, however, we note that one can also obtain a similar expression that puts the hops infinitesimally before $t = 0$, as was done in Eq. S38. Figures S2 and S3 show numerically that this is equivalent to the direct population dynamics used in the main text. The numerical methodology used to calculate these results is discussed in the next section.

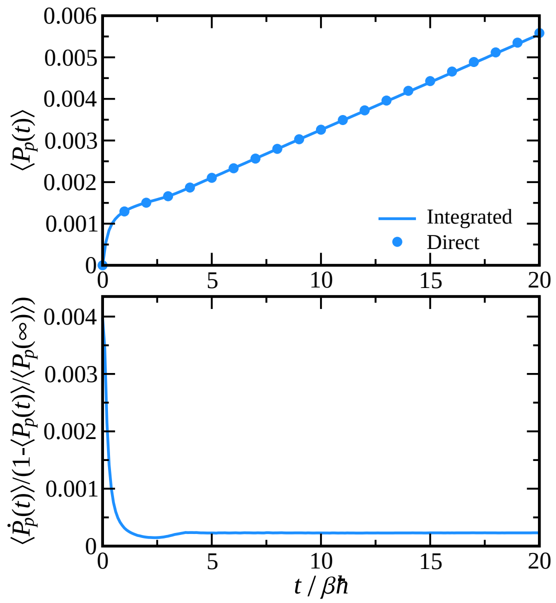

Figure S2: Demonstration of the MASH flux-correlation formalism. Upper panel: comparison of direct population dynamics (Eq. S15) and the cumulative integral of the flux-correlation formalism (Eq. S42) for the symmetric, $\beta\varepsilon = 0$, spin-boson model with $\log_{10}(\beta\Delta) = -1$, $\beta\hbar\Omega = 1/4$, $\gamma = \Omega$, and $\beta\Lambda = 12$. Lower panel: generalised flux-correlation function calculated using the flux-correlation formalism (Eq. S42) the long-time limit of which defines the rate constant. All results use the same spin-boson model, and the general definition of reactants and products.

Numerical implementation of flux-correlation formalism.

Position based definition (normal regime).

With some straightforward algebraic manipulations, Eq. S27 can be decomposed in the usual Bennett–Chandler form[S6] as follows

$$\begin{aligned}\langle \dot{P}_p(t)\rangle &= -\frac{\mathrm{tr}\left[e^{-\beta E}\dot{Q}\delta(Q-Q^\ddagger)|S_z(t)|P_r(t)\right]}{\mathrm{tr}\left[e^{-\beta E}\delta(Q-Q^\ddagger)\right]}\frac{\mathrm{tr}\left[e^{-\beta E}\delta(Q-Q^\ddagger)\right]}{\mathrm{tr}\left[e^{-\beta E}h(-(Q-Q^\ddagger))|S_z|\right]} \\ &= -\left\langle \dot{Q}|S_z(t)|P_r(t)\right\rangle_{Q(0)=Q^\ddagger}\frac{2\,\mathrm{tr}\left[(e^{-\beta H_+}+e^{-\beta H_-})\delta(Q-Q^\ddagger)\right]}{\mathrm{tr}\left[(e^{-\beta H_+}+e^{-\beta H_-})h(-(Q-Q^\ddagger))\right]} \\ &= -\left\langle \dot{Q}|S_z(t)|P_r(t)\right\rangle_{Q(0)=Q^\ddagger}\frac{2\left\langle\delta(Q-Q^\ddagger)\right\rangle_{\mathrm{MF}}}{\langle h(-(Q-Q^\ddagger))\rangle_{\mathrm{MF}}},\end{aligned} \tag{S43}$$

where $\langle\dots\rangle_{Q(0)=Q^\ddagger}$ indicates an average over the distribution

$$\rho_{Q(0)=Q^\ddagger}(\boldsymbol{p},\boldsymbol{q},\boldsymbol{S}) = \frac{e^{-\beta E(\boldsymbol{p},\boldsymbol{q},\boldsymbol{S})}\delta(Q-Q^\ddagger)}{\mathrm{tr}\left[e^{-\beta E(\boldsymbol{p},\boldsymbol{q},\boldsymbol{S})}\delta(Q-Q^\ddagger)\right]}, \tag{S44}$$

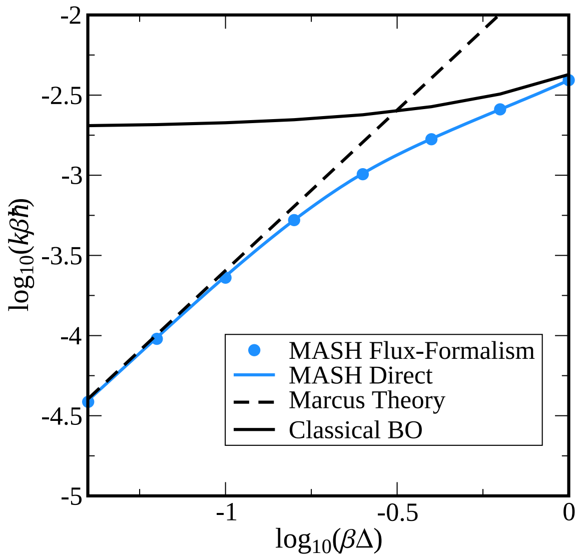

Figure S3: Log-log plot of the rate against the diabatic coupling, for symmetric, $\beta\varepsilon = 0$, spin-boson model, with $\beta\hbar\Omega = 1/4$, $\gamma = \Omega$ and $\beta\Lambda = 12$. Direct MASH rates were calculated from the slope of $\langle P_p(t)\rangle$, at the plateau time, between $t = 10\beta\hbar$ and $t = 20\beta\hbar$. Results from the flux-formulation were calculated using Eq. S42.

and $\langle \dots \rangle_{\mathrm{MF}}$ indicates an average over the thermal "Mean-Field" distribution

$$\rho_{\mathrm{MF}}(\boldsymbol{p},\boldsymbol{q}) = \frac{e^{-\beta H_+} + e^{-\beta H_-}}{\mathrm{tr}[e^{-\beta H_+} + e^{-\beta H_-}]}. \tag{S45}$$

Note that in this form, it is assumed that S_z is sampled with the correct Boltzmann weights. Alternatively, we can sample $\boldsymbol{S}$ uniformly from the Bloch sphere and write

$$\langle \dot{P}_p(t)\rangle = -\left\langle \frac{2e^{-\beta E}}{e^{-\beta H_+} + e^{-\beta H_-}} \dot{Q}|S_z(t)|P_r(t) \right\rangle_{Q(0)=Q^\ddagger,\mathrm{MF}} \frac{2\left\langle \delta(Q-Q^\ddagger)\right\rangle_{\mathrm{MF}}}{\langle h(-(Q-Q^\ddagger))\rangle_{\mathrm{MF}}}, \tag{S46}$$

where $\langle \dots \rangle_{Q(0)=Q^\ddagger,\mathrm{MF}}$ indicates an average over the distribution

$$\rho_{Q(0)=Q^\ddagger,\mathrm{MF}}(\boldsymbol{p},\boldsymbol{q}) = \frac{(e^{-\beta H_+} + e^{-\beta H_-})\delta(Q-Q^\ddagger)}{\mathrm{tr}[(e^{-\beta H_+} + e^{-\beta H_-})\delta(Q-Q^\ddagger)]}. \tag{S47}$$

Adiabatic state defintion (inverted regime).

We can evaluate Eq. S37 using a modified Bennett–Chandler procedure. We start by noting that the presence of the derivative coupling in

$$\dot{S}_z = -\sum_j \frac{2d_j(\boldsymbol{q})p_j}{m_j} S_x \tag{S48}$$

means that trajectories starting in regions of high derivative coupling will dominate. Ideally, one should therefore incorporate this into the sampling. Practically for our purposes, the sampling of the nuclear positions and momenta can be done from an as yet undefined distribution

$$\rho_{\text{samp}}(\boldsymbol{p}, \boldsymbol{q}) = \frac{e^{-\beta H_{\text{samp}}(\boldsymbol{p},\boldsymbol{q})}}{\text{tr}\left[e^{-\beta H_{\text{samp}}(\boldsymbol{p},\boldsymbol{q})}\right]}. \tag{S49}$$

The first term of Eq. S37 can be written in terms of averages over this distribution as

$$\begin{aligned}
&\frac{\text{tr}\left[e^{-\beta E}\delta_+(S_z)\dot{S}_z h(S_z(t))|S_z(t)|\right]}{\text{tr}\left[e^{-\beta E}h(S_z)|S_z|\right]} \\
&\qquad = \left\langle e^{-\beta(E-H_{\text{samp}})}\dot{S}_z h(S_z(t))|S_z(t)|\right\rangle_{\text{samp},S_z=0^+} \frac{\text{tr}\left[e^{-\beta H_{\text{samp}}}\delta_+(S_z)\right]}{\text{tr}\left[e^{-\beta E}h(S_z)|S_z|\right]}.
\end{aligned} \tag{S50}$$

The integrals over the spin-sphere in the second term can be performed analytically to give

$$\begin{aligned}
\frac{\text{tr}\left[e^{-\beta H_{\text{samp}}}\delta_+(S_z)\right]}{\text{tr}\left[e^{-\beta E}h(S_z)|S_z|\right]} &= \frac{\text{tr}\left[e^{-\beta H_{\text{samp}}}\right]\int_{-1}^{1}\mathrm{d}S_z\,\delta_+(S_z)}{\text{tr}\left[e^{-\beta H_+}\right]\int_{-1}^{1}\mathrm{d}S_z\,h(S_z)|S_z|} \\
&= \frac{2}{\langle e^{-\beta(H_+-H_{\text{samp}})}\rangle_{\text{samp}}},
\end{aligned} \tag{S51}$$

such that overall we have

$$\frac{\text{tr}\left[e^{-\beta E}\delta_+(S_z)\dot{S}_z h(S_z(t))|S_z(t)|\right]}{\text{tr}\left[e^{-\beta E}h(S_z)|S_z|\right]} = \frac{2\left\langle e^{-\beta(E-H_{\text{samp}})}\dot{S}_z h(S_z(t))|S_z(t)|\right\rangle_{\text{samp},S_z=0^+}}{\langle e^{-\beta(H_+-H_{\text{samp}})}\rangle_{\text{samp}}}. \tag{S52}$$

For the term constrained initially to the lower adiabatic surface, we can follow the same steps to obtain the final equation for the derivative of the population as

$$\begin{aligned}\frac{\mathrm{d}}{\mathrm{d}t}\langle P_p^{(a)}(t)\rangle =&\frac{\left\langle e^{-\beta(E-H_{\mathrm{samp}})}\dot{S}_z h(S_z(t))|S_z(t)|\right\rangle_{\mathrm{samp},S_z=0^+}}{\langle e^{-\beta(H_+-H_{\mathrm{samp}})}\rangle_{\mathrm{samp}}}\\ &+\frac{\left\langle e^{-\beta(E-H_{\mathrm{samp}})}\dot{S}_z h\left(\mathit{\Delta} E_{\mathrm{hop}}\right) h(S_z(t))|S_z(t)|\right\rangle_{\mathrm{samp},S_z=0^-}}{\langle e^{-\beta(H_+-H_{\mathrm{samp}})}\rangle_{\mathrm{samp}}}.\end{aligned} \tag{S53}$$

Here we give the expression for the symmetric definition of the derivative, $\dot{P}_p^{(a)}$, however a similar expression can also be derived for Eq. S38. Note that sampling from the distribution with $S_z(0) = 0^+$ or $S_z(0) = 0^-$ is implemented in practice by just setting the initial S_z to a small floating point number, $\pm 10^{-10}$.

General definition (used in main paper).

Combining the ideas of the last two sections, we can arrive at a Bennett–Chandler scheme to efficiently compute the rate with the general dividing surface. For the first term in Eq. S42, this can be achieved by writing

$$\begin{aligned}&\frac{\mathrm{tr}\left[e^{-\beta E}\dot{Q}\delta(Q-Q^\ddagger)[h(S_z)-h(-S_z)]P_r^{(g)}(t)|S_z(t)|\right]}{\mathrm{tr}\left[e^{-\beta E}[h(-(Q-Q^\ddagger))h(-S_z)+h(Q-Q^\ddagger)h(S_z)]|S_z|\right]}\\ &=\left\langle \dot{Q}[h(S_z)-h(-S_z)]P_r^{(g)}(t)|S_z(t)|\right\rangle_{Q(0)=Q^\ddagger}\frac{2\,\mathrm{tr}\left[(e^{-\beta H_+}+e^{-\beta H_-})\delta(Q-Q^\ddagger)\right]}{\mathrm{tr}\left[e^{-\beta H_+}h(Q-Q^\ddagger)+e^{-\beta H_-}h(-(Q-Q^\ddagger))\right]}\\ &=\frac{2\left\langle \dot{Q}[h(S_z)-h(-S_z)]P_r^{(g)}(t)|S_z(t)|\right\rangle_{Q(0)=Q^\ddagger}\langle\delta(Q-Q^\ddagger)\rangle_{\mathrm{MF}}}{\left\langle \frac{e^{-\beta H_+}}{e^{-\beta H_+}+e^{-\beta H_-}}h(Q-Q^\ddagger)+\frac{e^{-\beta H_-}}{e^{-\beta H_+}+e^{-\beta H_-}}h(-(Q-Q^\ddagger))\right\rangle_{\mathrm{MF}}}.\end{aligned} \tag{S54}$$

Alternatively sampling $\boldsymbol{S}$ uniformly from the surface of a sphere, we have that

$$\begin{aligned}&\left\langle \dot{Q}[h(S_z)-h(-S_z)]P_r^{(g)}(t)|S_z(t)|\right\rangle_{Q(0)=Q^\ddagger}\\ &\qquad=\left\langle \frac{2e^{-\beta E}}{e^{-\beta H_+}+e^{-\beta H_-}}\dot{Q}[h(S_z)-h(-S_z)]P_r^{(g)}(t)|S_z(t)|\right\rangle_{Q(0)=Q^\ddagger,\mathrm{MF}}.\end{aligned} \tag{S55}$$

For the second term and third term, we can follow the same approach as with the pure adiabatic

dividing surface. This gives

$$\frac{\operatorname{tr}\left[e^{-\beta E}[h(Q-Q^\ddagger)-h(-(Q-Q^\ddagger))]\delta_+(S_z)\dot{S}_z P_r^{(g)}(t)|S_z(t)|\right]}{2\operatorname{tr}\left[e^{-\beta E}[h(-(Q-Q^\ddagger))h(-S_z)+h(Q-Q^\ddagger)h(S_z)]|S_z|\right]} \\ = \frac{\left\langle e^{-\beta(E-H_{\mathrm{samp}})}[h(Q-Q^\ddagger)-h(-(Q-Q^\ddagger))]\dot{S}_z P_r^{(g)}(t)|S_z(t)|\right\rangle_{\mathrm{samp},S_z=0^+}}{\left\langle e^{-\beta(H_--H_{\mathrm{samp}})}h(-(Q-Q^\ddagger))+e^{-\beta(H_+-H_{\mathrm{samp}})}h(Q-Q^\ddagger)\right\rangle_{\mathrm{samp}}} \tag{S56}$$

and

$$\frac{\operatorname{tr}\left[e^{-\beta E}[h(Q-Q^\ddagger)-h(-(Q-Q^\ddagger))]\delta_-(S_z)\dot{S}_z h\left(\mathit{\Delta} E_{\mathrm{hop}}\right) P_r^{(g)}(t)|S_z(t)|\right]}{2\operatorname{tr}\left[e^{-\beta E}[h(-(Q-Q^\ddagger))h(-S_z)+h(Q-Q^\ddagger)h(S_z)]|S_z|\right]} \\ = \frac{\left\langle e^{-\beta(E-H_{\mathrm{samp}})}[h(Q-Q^\ddagger)-h(-(Q-Q^\ddagger))]h(\mathit{\Delta} E_{\mathrm{hop}})\dot{S}_z P_r^{(g)}(t)|S_z(t)|\right\rangle_{\mathrm{samp},S_z=0^-}}{\left\langle e^{-\beta(H_--H_{\mathrm{samp}})}h(-(Q-Q^\ddagger))+e^{-\beta(H_+-H_{\mathrm{samp}})}h(Q-Q^\ddagger)\right\rangle_{\mathrm{samp}}}. \tag{S57}$$

Here we have chosen to use the symmetric definition of $\frac{\mathrm{d}}{\mathrm{d}t}h(S_z(t))$ (Eq. S36). This means that hops can occur within the first time step, however we found that this did not present a significant numerical difficulty. We found that it was more important to sample the initial momenta such that for each $\boldsymbol{p}$, we also sampled a trajectory with $-\boldsymbol{p}$ at the same $\boldsymbol{S}$ and $\boldsymbol{q}$. We note that this could have also been achieved by using the asymmetric version, where hops are always infinitesimally before $t=0$, by appropriately rescaling the momenta. Finally in the present calculations, we used a very simple choice for H_{samp} corresponding to sampling from the reactant diabatic potential origin, shifted to the diabatic crossing seam

$$H_{\mathrm{samp}}(\boldsymbol{p},\boldsymbol{q})=\sum_{j=1}^{f}\frac{p_j^2}{2m_j}+U_0(\boldsymbol{q}-\boldsymbol{q}_{\mathrm{shift}}) \tag{S58}$$

with $\boldsymbol{q}_{\mathrm{shift}}=(Q^\ddagger,0,0,0,\dots)$. This is not the optimal choice, as it does not take into account the narrowing of the derivative coupling as $\Delta\to 0$, however it was sufficient for the present purpose.

Additional Results.

In the following, we include results of additional systems, that support the conclusions of the main text.

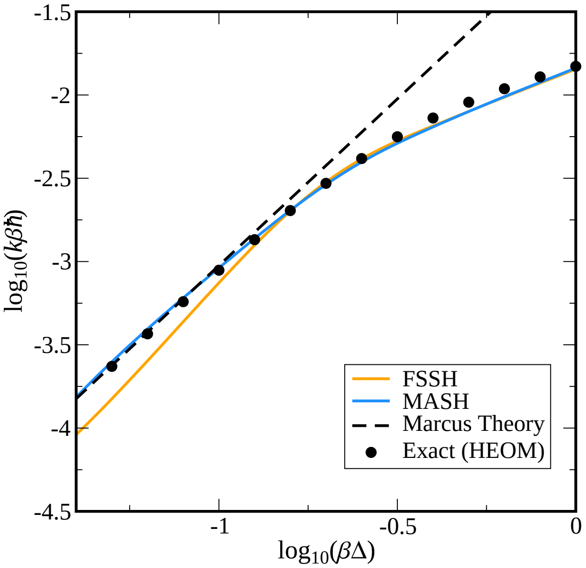

Figure S4: Log-log plot of the rate against the diabatic coupling, for an asymmetric, $\beta\varepsilon = 3$, spin-boson model, with $\beta\hbar\Omega = 1/4$, $\gamma = \Omega$ and $\beta\Lambda = 12$. FSSH and MASH rates were calculated from the slope of $\langle P_p(t)\rangle$, at the plateau time, between $t = 10\beta\hbar$ and $t = 20\beta\hbar$. This shows essentially the same behaviour as seen for the symmetric system in Fig. 1 of the main text.

In Fig. S4, we show that the qualitative behaviour is unchanged from Fig. 1 of the main text when one adds a small bias to products, $\beta\varepsilon = 3$.

In Fig. S5, we consider a system which is equivalent to that considered in Fig. 1 of the main text, except that it is in the overdamped $\gamma = 4\Omega$ rather than underdamped regime. This illustrates that the overcoherence problem is less significant at high friction.

In Fig. S6, we show the results of including the simple decoherence correction on FSSH. These were left out of the main paper to avoid clutter. This illustrates that unlike for MASH, the simple decoherence correction is unable to fix FSSH.

In Fig. S7, we consider a system with a large reorganisation energy, $\beta\Lambda = 60$, and high friction, $\gamma = 32\Omega$. This illustrates the utility of the flux-correlation formalism, as direct calculation of the rates would be prohibitively expensive at the smallest values of Δ considered.

In Fig. S8, we consider a system the same system as Fig. 1 of the main text. This figure illustrates that the added decoherence correction does not affect the ability of MASH to predict the correct Δ^2 behavior.

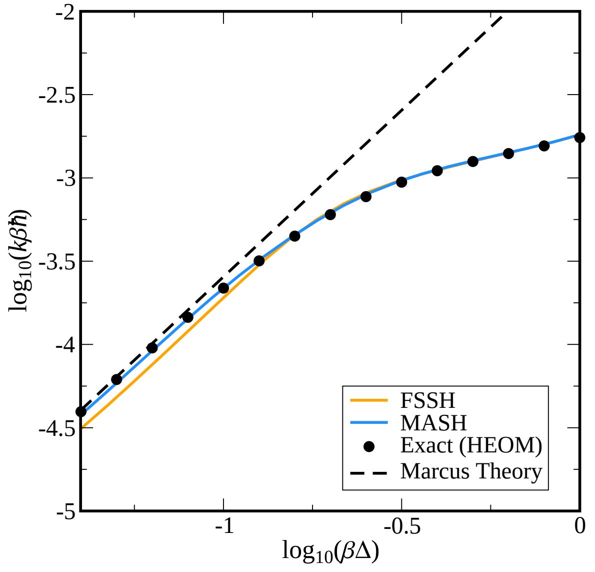

Figure S5: Log-log plot of the rate against the diabatic coupling, for a symmetric, $\beta\varepsilon = 0$, spin-boson model, with $\beta\hbar\Omega = 1/4$, and $\beta\Lambda = 12$ in the overdamped regime $\gamma = 4\Omega$. FSSH and MASH rates were calculated from the slope of $\langle P_p(t)\rangle$, at the plateau time, between $t = 10\beta\hbar$ and $t = 20\beta\hbar$.

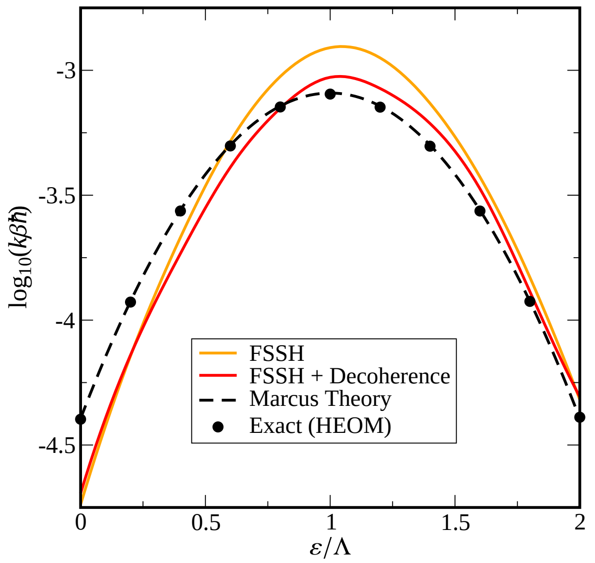

Figure S6: Logarithmic plot of the rate against reaction driving force, showing the famous Marcus turn over behaviour for a spin-boson model with weak diabatic coupling, $\log_{10}(\beta\Delta) = -7/5$, $\beta\hbar\Omega = 1/4$, $\gamma = \Omega$, and $\beta\Lambda = 12$. FSSH rates were calculated from the slope of $\langle P_p(t)\rangle$, at the plateau time, between $t = 10\beta\hbar$ and $t = 20\beta\hbar$. To obtain the red line, the simple gap-based decoherence correction was applied whenever $V_+ - V_- > 4k_\mathrm{B}T$.

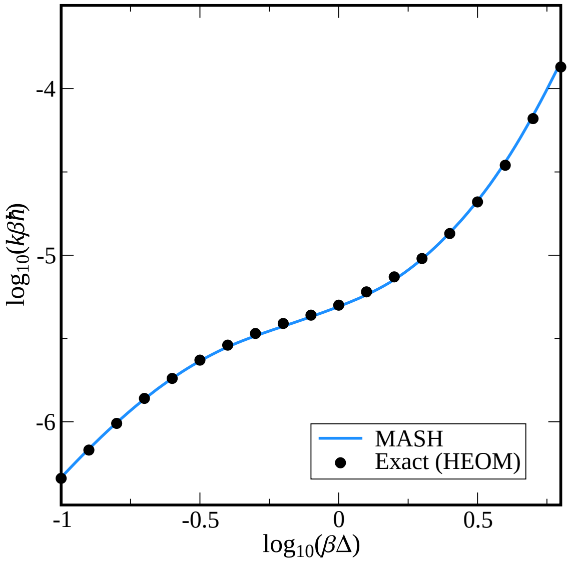

Figure S7: Log-log plot of the rate against the diabatic coupling, for an asymmetric, $\varepsilon = \Lambda/4$, spin-boson model, with $\beta\hbar\Omega = 1/2$, $\gamma = 32\Omega$ and $\beta\Lambda = 60$. MASH rates were calculated using the flux-correlation formalism, and exact HEOM results were taken from Ref. S3.

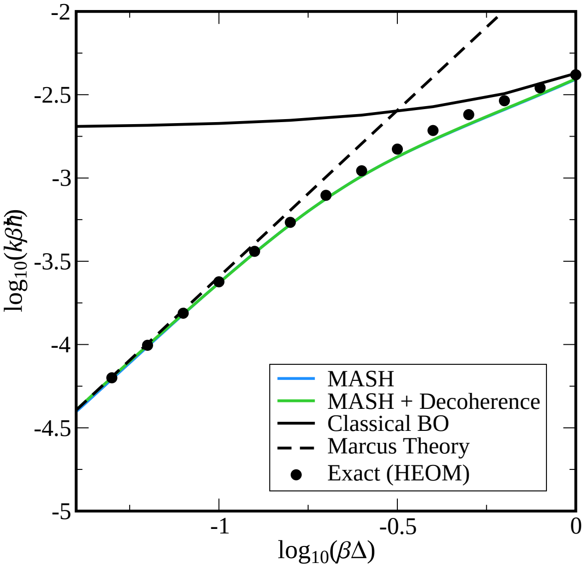

Figure S8: Log-log plot of the rate against the diabatic coupling for a symmetric, $\beta\varepsilon = 0$, spin-boson model, with $\beta\hbar\Omega = 1/4$, $\gamma = \Omega$ and $\beta\Lambda = 12$. MASH rates were calculated from the slope of $\langle P_p(t)\rangle$ at the plateau time, between $t = 10\beta\hbar$ and $t = 20\beta\hbar$. Equivalent to Fig. 1 of main text showing that decoherence correction does not change the MASH result.

References

(S1) Mannouch, J. R.; Richardson, J. O. A mapping approach to surface hopping. *J. Chem. Phys.* **2023**, *158*, 104111.

(S2) Craig, I. R.; Thoss, M.; Wang, H. Proton transfer reactions in model condensed-phase environments: Accurate quantum dynamics using the multilayer multiconfiguration time-dependent Hartree approach. *J. Chem. Phys.* **2007**, *127*, 144503.

(S3) Lawrence, J. E.; Fletcher, T.; Lindoy, L. P.; Manolopoulos, D. E. On the calculation of quantum mechanical electron transfer rates. *J. Chem. Phys.* **2019**, *151*, 114119.

(S4) Chandler, D. *Introduction to Modern Statistical Mechanics*; Oxford University Press: New York, 1987.

(S5) Amati, G.; Mannouch, J. R.; Runeson, J. E.; Richardson, J. O. Detailed balance in mixed quantum–classical mapping approaches. arXiv:2309.04686 [quant–ph].

(S6) Frenkel, D.; Smit, B. *Understanding Molecular Simulation*, 2nd ed.; Elsevier: San Diego, 1996.
